# Supplementary material for: Early feasibility study with an implantable near-infrared spectroscopy sensor for glucose, ketones, lactate and ethanol
Source: PLoS One. 2024 May 3;19(5):e0301041. doi: 10.1371/journal.pone.0301041 (PMC11068174; doi:10.1371/journal.pone.0301041)
Supplement: S1 File — (PDF) [file pone.0301041.s002.pdf]

UZA / Wilrijkstraat 10 / 2650 Edegem
Parking via Drie Eikenstraat 655
Tel +32 3 821 30 00
www.uza.be / BE0874.619.603

Universiteit
Antwerpen
Ethical committee

Prof. dr. C. De Block

Endocrinologie, diabetologie en metabole ziekten

PRESIDENT
Prof. dr. Peter Michielsen

SECRETARIAAT
tel: 03 821 38 97

An open label, interventional prospective study om to evaluate safety and short-term tissue integration of the YANG SENSOR (EDGE 001284) (ProtocolNr: IND007)

Belgian Registration number: 83002020000190

date	ons kenmerk	contactpersoon
2/11/2020	20/40/530	Secretariaat Ethisch Comité<br>ethisch.comite@uza.be

FINAL APPROVAL

Dear Colleague,

The ethical commitee of Antwerp Unviversity Hospital and Antwerp University hererby confirms dat the abovementioned study conforms to the requirements in the law of May 7, 2004 and gave a favorable advice on Nov 2nd, 2020.

Following attachments were approved by the ethical committe according to the IHC-GCP guidelines:

- Documentation of "no-fault" insurance, adapted version dd. October 15, 2020
  HDI, policy nr. 309/08294992-3005, expiry date 31/03/2021
- CV Principal Investigator UZA dd. 16/09/2020
  Prof. Dr. Chr. De Block, signed
- Informed consent form NL adapted version dd. 29/10/2020
  Version 3.0
- Protocol dd. 17/09/2020
  Version 1.0
- Protocol - Synopsis dd. 17/09/2020
  Version 1.0
- Investigator's Brochure dd. 18/09/2020
  Versie 1.0
- Case report form(s) dd. 17/09/2020
  Design version 1
- Miscellaneous
  Declaration of conformity dd. 18/09/2020

UZA / Wilrijkstraat 10 / 2650 Edegem
Parking via Drie Eikenstraat 655
Tel +32 3 821 30 00
www.uza.be / BE0874.619.603

Universiteit
Antwerpen

date
2/11/2020

reference
20/40/530

contactpersoon
Secretariaat Ethisch Comité
ethisch.comite@uza.be

This approval remains valid up to a year after the approval date. We request to be notified when the first participant is included, when and why the study is stopped (prematurely) or was never initiated.
If the study is still ongoing after a year, we expect a follw-up report describing any incidents that may have occurred.
Finally, we point out that for studies conducted at UZA, serious adverse events must be reported via the incident notification system of the hospital.

Met vriendelijke With kind regards,

Prof. dr. P. Michielsen
President Ethical Committee

CC: FAGG - Research & Development Department, Victor Hortaplein 40, bus 40 - 1060 Brussel

UZA / Wilrijkstraat 10 / 2650 Edegem
Parking via Drie Eikenstraat 655
Tel +32 3 821 30 00
www.uza.be / BE0874.619.603

Universiteit Antwerpen

datum
2/11/2020

ons kenmerk
20/40/530

contactpersoon
Secretariaat Ethisch Comité
ethisch.comite@uza.be

Composition of the ethical committee since 1/05/2020.

This study was discussed on the committee's meeting of 2/11/2020.

	Function	M/F	Present
President			
MICHIELSEN Peter	Voorzitter/Gastro-enteroloog	M	+
Vice President			
CRAS Patrick	Ondervoorzitter/Neuroloog	M	+
IEVEN Greet	Ondervoorzitter/Klinisch Bioloog	F	+
Members			
BLAUMEISER Bettina	Medisch geneticus	F	+
DE BAETSELIER Elyne	Verpleegkundige	F	+
FIERENS Lina	Verpleegkundige	F	+
HENS Kristien	Ethicus	F	-
KWAKKEL-VAN ERP Hanneke	Pneumoloog	F	-
MICHIELS Barbara	Huisarts	F	+
PAELINCK Bernard	Cardiochirurg	M	-
SCHOETERS Veerle	Verpleegkundige	F	+
VAN DE WIELE Miranda	Patiëntenbegeleiding	F	-
VAN DEN BRANDE Jan	Oncoloog	M	-
VAN DEN EEDE Filip	Psychiater	M	-
VAN DYCK Pieter	Radioloog	M	-
VAN PRAAG Dominique	Psychologe	F	-
VANSWEEVELT Thierry	Jurist	M	-
VERLOOY Joris	Pediater	M	-
WYERS Griet	Apotheker	F	+
Toegevoegde leden UA			
BORTIER Hilde	Arts, emerita hoogleraar UA	F	-
DE MEESTER Ingrid	Farmacoloog / Onderzoeker UA	F	+
GRANAAS Kristina	Medewerkster Dep. Onderzoek	F	-
Uitgenodigde Experten			
IDES Kris	Kinesitherapeut	M	-
LUYTEN Leon	Arts in het beheer van gezondheidsgegevens	M	-
MICHIELSENS Inge	Jurist	F	-
MOONS Pieter	Coördinator bio- en weefselbank	M	-
VAN DE WILDEBERGH Daphné	Jurist	F	-
VAN HONSTE Guy	Patiëntenvertegenwoordiger	M	+

The Ethics Committee states that no individual member of the Ethics Committee who may have an affiliation with the study or sponsor, has voted in the deliberations for this trial.

The Ethics Committee states that it is organised and operates according to the ICH/GCP guidelines, the applicable laws and regulations, and their own written operating procedures.
