# Supplementary material for: Early feasibility study with an implantable near-infrared spectroscopy sensor for glucose, ketones, lactate and ethanol
Source: PLoS One. 2024 May 3;19(5):e0301041. doi: 10.1371/journal.pone.0301041 (PMC11068174; doi:10.1371/journal.pone.0301041)
Supplement: S2 File — (PDF) [file pone.0301041.s003.pdf]

UZA / Wilrijkstraat 10 / 2650 Edegem
Parking via Drie Eikenstraat 655
Tel +32 3 821 30 00
www.uza.be / BE0874.619.603

Universiteit
Antwerpen
ETHISCH COMITE

Prof. dr. C. De Block

Endocrinologie, diabetologie en metabole ziekten

VOORZITTER
Prof. dr. Peter Michielsen

SECRETARIAAT
tel: 03 821 38 97

Een open label, interventionele prospectieve studie om de veiligheid en de korte termijn weefsel integratie van de YANG SENSOR te onderzoeken (EDGE 001284) (ProtocolNr: IND007)

Belgisch Registratienummer: B3002020000190

datum	ons kenmerk	contactpersoon
2/11/2020	20/40/530	Secretariaat Ethisch Comité<br>ethisch.comite@uza.be

DEFINITIEF GUNSTIG ADVIES

Geachte Collega,

Het Ethisch Comité van het Universitair Ziekenhuis Antwerpen en de Universiteit Antwerpen bevestigt dat bovenvermelde studie voldoet aan de criteria gesteld in de wet van 7 mei 2004 en geeft een gunstig advies dd. 2/11/2020.

De volgende bijlagen werden volgens de ICH-GCP richtlijnen door het Ethisch Comité goedgekeurd:

- Bewijs van "no-fault" verzekering aangepaste versie dd. 15/10/2020
  HDI, polisnr. 309/08294992-30056, exp date 31/03/2021
- CV Onderzoeker UZA dd. 16/09/2020
  Prof. Dr. Chr. De Block, signed
- Informatie- en toestemmingsformulier NL aangepaste versie dd. 29/10/2020
  Versie 3.0
- Protocol dd. 17/09/2020
  Versie 1.0
- Protocol - Samenvatting dd. 17/09/2020
  Versie 1.0
- Investigator's Brochure dd. 18/09/2020
  Versie 1.0
- Case report form(s) dd. 17/09/2020
  Design version 1
- Diverse
  Declaration of conformity dd. 18/09/2020

UZA / Wilrijkstraat 10 / 2650 Edegem
Parking via Drie Eikenstraat 655
Tel +32 3 821 30 00
www.uza.be / BE0874.619.603

Universiteit Antwerpen

datum
2/11/2020

ons kenmerk
20/40/530

contactpersoon
Secretariaat Ethisch Comité
ethisch.comite@uza.be

Deze goedkeuring is geldig tot een jaar na bovenvermelde datum. Wij verzoeken u ons te melden wanneer de eerste deelnemer werd geïncludeerd, wanneer en waarom de studie (vroegtijdig) werd stopgezet of nooit werd opgestart.
Indien de studie nog loopt na een jaar verwachten we een follow-up rapport waarin eventuele voorvallen worden gemeld.
Tot slot wijzen we er op dat voor in het UZA lopende studies de ernstige ongewenste voorvallen dienen gerapporteerd via het incidentenmeldingssysteem.

Met vriendelijke groeten,

Prof. dr. P. Michielsen
Voorzitter Ethisch Comité

Cc: FAGG - Research & Development Department, Victor Hortaplein 40, bus 40 - 1060 Brussel

UZA / Wilrijkstraat 10 / 2650 Edegem
Parking via Drie Eikenstraat 655
Tel +32 3 821 30 00
www.uza.be / BE0874.619.603

Universiteit
Antwerpen

datum
2/11/2020

ons kenmerk
20/40/530

contactpersoon
Secretariaat Ethisch Comité
ethisch.comite@uza.be

Samenstelling Ethisch Comité sinds 1/05/2020.
Deze studie werd besproken op vergadering van 2/11/2020.

	Functie	M/V	Aanwezig
Voorzitter			
MICHIELSEN Peter	Voorzitter/Gastro-enteroloog	M	+
Ondervoorzitter			
CRAS Patrick	Ondervoorzitter/Neuroloog	M	+
IEVEN Greet	Ondervoorzitter/Klinisch Bioloog	F	+
Leden EC UZA			
BLAUMEISER Bettina	Medisch geneticus	F	+
DE BAETSELIER Elyne	Verpleegkundige	F	+
FIERENS Lina	Verpleegkundige	F	+
HENS Kristien	Ethicus	F	-
KWAKKEL-VAN ERP Hanneke	Pneumoloog	F	-
MICHIELS Barbara	Huisarts	F	+
PAELINCK Bernard	Cardiochirurg	M	-
SCHOETERS Veerle	Verpleegkundige	F	+
VAN DE WIELE Miranda	Patiëntenbegeleiding	F	-
VAN DEN BRANDE Jan	Oncoloog	M	-
VAN DEN EEDE Filip	Psychiater	M	-
VAN DYCK Pieter	Radioloog	M	-
VAN PRAAG Dominique	Psychologe	F	-
VANSWEEVELT Thierry	Jurist	M	-
VERLOOY Joris	Pediater	M	-
WYERS Griet	Apotheker	F	+
Toegevoegde leden UA			
BORTIER Hilde	Arts, emerita hoogleraar UA	F	-
DE MEESTER Ingrid	Farmacoloog / Onderzoeker UA	F	+
GRANAAS Kristina	Medewerkster Dep. Onderzoek	F	-
Uitgenodigde Experten			
IDES Kris	Kinesitherapeut	M	-
LUYTEN Leon	Arts in het beheer van gezondheidsgegevens	M	-
MICHIELSENS Inge	Jurist	F	-
MOONS Pieter	Coördinator bio- en weefselbank	M	-
VAN DE WILDEBERGH Daphné	Jurist	F	-
VAN HONSTE Guy	Patiëntenvertegenwoordiger	M	+

The Ethics Committee states that no individual member of the Ethics Committee who may have an affiliation with the study or sponsor, has voted in the deliberations for this trial.
The Ethics Committee states that it is organised and operates according to the ICH/GCP guidelines, the applicable laws and regulations, and their own written operating procedures.
