# Supplementary material for: Early feasibility study with an implantable near-infrared spectroscopy sensor for glucose, ketones, lactate and ethanol
Source: PLoS One. 2024 May 3;19(5):e0301041. doi: 10.1371/journal.pone.0301041 (PMC11068174; doi:10.1371/journal.pone.0301041)
Supplement: S4 File — (PDF) [file pone.0301041.s005.pdf]

The GLOW Study

**Study Number**	IND007
**Protocol Version/Date**	2.0 / 30 March 2021
**Sponsor**	**Indigo Diabetes N.V.**,<br>Bollebergen 2B box 5,<br>9000 Gent, Belgium<br>https://indigomed.com/
**Protocol Authors**	**Karolina Janikowska, PhD**<br>Clinical, Quality and Regulatory Affairs Consultant<br>Medidee Services SA<br>Chemin de Rovéréaz 5<br>CH-1012 Lausanne, Switzerland<br>Karolina.janikowska@medidee.com<br>Mobile: +41 76 219 37 78<br>**Delphine Huser, PhD**<br>Clinical, Quality and Regulatory Affairs Consultant<br>Medidee Services SA<br>Chemin de Rovéréaz 5<br>CH-1012 Lausanne, Switzerland<br>Delphine.huser@medidee.com<br>Mobile: + 41 79 779 18 08
**Protocol Reviewers**	**Dr. med. Gijs Klarenbeek**<br>Vice President Clinical Affairs<br>Indigo Diabetes N.V., Bollebergen 2B box 5,<br>9000 Gent, Belgium<br>gijs.klarenbeek@indigomed.com<br>Mobile : +32 479 98 64 58
**Principal Investigator**	**Prof. Dr. med. Christophe De Block**<br>Head of Endocrinology, Diabetology and Metabolism;<br>Antwerp University Hospital (UZA)<br>Wilrijkstraat 10<br>2650 Edegem, Belgium<br>diabetologie@uza.be or christophe.deblock@uza.be<br>Mobile: +32 3 821 32 75
**Clinical Research Organizations (CROs)**	**Writing of clinical trial application dossier**<br>**Medidee Services SA**<br>Chemin de Rovéréaz 5<br>CH-1012 Lausanne, Switzerland

CONFIDENTIAL - THIS DOCUMENT CONTAINS CONFIDENTIAL AND PROPRIETARY INFORMATION. THIS DOCUMENT MAY NOT BE REPRODUCED, USED, OR DISCLOSED TO ANY THIRD PARTY WITHOUT PRIOR WRITTEN CONSENT.

	www.medidee.com<br>**Monitoring activities and eCRF design**<br>**TRIUM Clinical Consulting, NV**<br>Baron Opsomerlaan 32<br>2500 Lier

Confidentiality Statement

The information in this clinical investigation plan is for the CRO, sponsor, investigator and staff, ethics committee and health authorities. It may not be disclosed to third parties without written authorization from Indigo Diabetes N.V., except to obtain informed consent from persons receiving the study treatment. Once signed, the terms of this clinical investigation plan are binding for all parties.

Indigo Diabetes N.V
Bollebergen 2B box 5,
9000 Gent, Belgium
https://indigomed.com/

Signature Page

This clinical investigation plan (CIP) was subject to a critical review, and has been approved by Indigo Diabetes N.V. The contained information is consistent with:

- The current risk/benefit evaluation of the device,
- The moral, ethical and scientific principles governing clinical research as set out in the Declaration of Helsinki in its current version,
- The principles of Good clinical practice guidelines as applicable for medical devices in their current version: ISO 14155:2020: Clinical investigation of medical devices for human subjects — Good clinical practice
- Council Directive 90/385/EEC of 20 June 1990 on the approximation of the laws of the Member States relating to active implantable medical devices (referred here after as AIMDD- Active Implantable Medical Device Directive)

SPONSOR:

Dr. Med. Gijs Klarenbeek
Vice President Clinical Affairs

Apr 8, 2021 Gijs Klarenbeek
Gijs Klarenbeek (Apr 8, 2021 22:32 GMT+2)
Date/Signature

M Jongboer
VP Quality

Apr 8, 2021
Date/Signature

INVESTIGATOR:

Prof. Dr. med. Christophe De Block
Head of Endocrinology, Diabetology and Metabolism;
Antwerp University Hospital (UZA)

Apr 12, 2021 Christophe De Block
Christophe De Block (Apr 12, 2021 09:36 GMT+2)
Date/Signature

CONFIDENTIAL - THIS DOCUMENT CONTAINS CONFIDENTIAL AND PROPRIETARY INFORMATION. THIS DOCUMENT MAY NOT BE REPRODUCED, USED, OR DISCLOSED TO ANY THIRD PARTY WITHOUT PRIOR WRITTEN CONSENT.

Tables of Contents

1 Introduction 6
1.1 Purpose of this Document 6
1.2 Study Sponsor 6
1.3 Investigators 6
1.4 Investigation Site 6
1.5 Synopsis of the Clinical Investigation Plan 7
2 List of Abbreviations 11
3 Investigational Device 12
3.1 Summary Description of the Investigational Device 12
3.2 Principle and Mechanism of Action 12
3.3 Investigational Device Traceability and Accountability 13
4 Justification for the Design of the Clinical Investigation 14
4.1 Introduction 14
4.2 Investigational Device and Current Technology 14
5 Risk and Benefits of the Study Device and Investigation 15
5.1 Anticipated Clinical Benefits 15
5.2 Risks Associated with this Investigations and Residuals Risks 16
6 Study Objectives and Endpoints 16
6.1 Study Objectives 16
6.2 Study Endpoints and Variables 16
7 Design of the Study 18
7.1 General 18
7.2 Planned Study Steps 19
7.3 Test Subjects 19
7.4 Procedures 21
7.5 Adverse Events, Adverse Device Effects and Device Deficiencies 32
7.6 Suspension or Premature Termination of the Clinical Investigation 40
7.7 Monitoring Plan 40
8 Statistical Considerations 40
8.1 Justification of Sample Size 40
9 Data Management 41
9.1 Data Management Plan 41
9.2 Data Collection and Entry 41
9.3 Data Verification 41
9.4 Data Cleaning 41
9.5 Data Analysis 42
9.6 Data Retention 42
9.7 Protection of Patient's Privacy 42
10 Ethical and Legal Aspects 42
11 Data Quality Assurance (Auditing) 42
12 Amendments to this Study Plan 42
13 Deviations from the Clinical Investigation Plan 43

CONFIDENTIAL - THIS DOCUMENT CONTAINS CONFIDENTIAL AND PROPRIETARY INFORMATION. THIS DOCUMENT MAY NOT BE REPRODUCED, USED, OR DISCLOSED TO ANY THIRD PARTY WITHOUT PRIOR WRITTEN CONSENT.

Indigo Diabetes N.V
Bollebergen 2B box 5,
9000 Gent, Belgium
https://indigomed.com/

14 Financial Plan and Costs 43
15 Publication Policy 43
16 Conflict of Interest 44
17 Statement of Compliance 44
18 Bibliography 44
APPENDIX I: Revision History 46

CONFIDENTIAL - THIS DOCUMENT CONTAINS CONFIDENTIAL AND PROPRIETARY INFORMATION. THIS DOCUMENT MAY NOT BE REPRODUCED, USED, OR DISCLOSED TO ANY THIRD PARTY WITHOUT PRIOR WRITTEN CONSENT.

1 Introduction

1.1 Purpose of this Document

The Purpose of this Clinical Investigation Plan is to provide information related to the planned clinical study. This document states the rationale, objectives, design and proposed analysis, methodology, monitoring, conduct and record keeping related to the clinical investigation of the investigational device: YANG SYSTEM; composed of YANG SENSOR and YANGEXD (the external device and remote control).

1.2 Study Sponsor

Indigo Diabetes N.V.,
Bollebergen 2B box 5,
9000 Gent, Belgium
Represented by David Stocker, MD

1.3 Investigators

1.3.1 Principal Investigator

Prof. Dr. med. Christophe De Block
Head of Endocrinology, Diabetology and Metabolism;
Antwerp University Hospital (UZA)
Wilrijkstraat 10
2650 Edegem, Belgium
diabetologie@uza.be or christophe.deblock@uza.be
Mobile +32 3 821 32 75

1.3.2 Co-Investigators

Prof. Tadej Battelino, M.D., Ph.D.
Head, Dept. of Endocrinology, Diabetes & Metabolism
UCH, Professor, Faculty of Medicine, University of Ljubljana
Bohoriceva 20
1000 Ljubljana, Slovenia
tadej.battelino@mf.uni-lj.si
Mobile +386 40 218 202

1.4 Investigation Site

The study is a monocentric trial and will be conducted on the following site:

Antwerp University Hospital (UZA)
Wilrijkstraat 10
2650 Edegem, Belgium
Phone: +32 3 821 30 00
https://www.uza.be

CONFIDENTIAL - THIS DOCUMENT CONTAINS CONFIDENTIAL AND PROPRIETARY INFORMATION. THIS DOCUMENT MAY NOT BE REPRODUCED, USED, OR DISCLOSED TO ANY THIRD PARTY WITHOUT PRIOR WRITTEN CONSENT.

1.5 Synopsis of the Clinical Investigation Plan

**Study Title**	The GLOW Study
**Project Number**	IND007
**Sponsor**	Indigo Diabetes N.V.,<br>Bollebergen 2B box 5, 9000 Gent, Belgium
**Study Manager**	Juan Ordonez PhD,<br>Director Implant Technology and Scientific Affairs,<br>Indigo Diabetes N.V.,<br>Bollebergen 2B box 5, 9000 Gent, Belgium
**Principal Investigator and Related Study Site**	Prof. Dr. med. Christophe De Block<br>Head of Endocrinology, Diabetology and Metabolism<br>Antwerp University Hospital (UZA)<br>Wilrijkstraat 10, 2650 Edegem, Belgium
**Investigational Device**	YANG SYSTEM, composed of implantable YANG SENSOR and YANGEXD external device
**Class of Investigational Device**	YANG SENSOR is an active implantable device (as per AIMDD)
**Preclinical Testing**	All relevant preclinical investigations which are necessary in the context of the planned clinical investigation have been performed successfully and referred to in the Investigator’s Brochure (IB).
**Mode and Application of the Investigational Device**	The YANG SENSOR is an active implantable device, that is intended to be implanted in the subcutaneous abdominal tissue, approximately 10mm below the skin. The SENSOR is battery powered.<br>The battery is wirelessly recharged through the skin by the YANGEXD, using a charging coil (‘Donut’) applied to the skin with an adhesive patch at the level of the SENSOR. The ‘Donut’ also receives data from the SENSOR.
**Name and Type of Comparator**	No direct comparator will be used in the study. While collected by the investigational device, the glucose, ketones and lactate values will be also measured in blood samples during the measurement visits using standard techniques.<br>In addition, the glucose values will be analysed by a commercially available CGM device (Dexcom G6).
**Study Design**	This is an open label, interventional, monocentric, prospective early feasibility study, designed to evaluate the safety of implant and short-term integration into the tissue of the device. In additions, the set up will enable data collection (that is, raw NIR spectra of ketones, glucose and lactate levels from subjects’ interstitial fluid), which will be used to develop the software algorithm to allow real-time measurements in a future version of the YANG SENSOR
**Study Duration**	Duration of the study per participant: 57 days<br>Duration of the overall study; first patient first visit (FPFV) to last patient last visit (LPLV): expected 4 months

CONFIDENTIAL - THIS DOCUMENT CONTAINS CONFIDENTIAL AND PROPRIETARY INFORMATION. THIS DOCUMENT MAY NOT BE REPRODUCED, USED, OR DISCLOSED TO ANY THIRD PARTY WITHOUT PRIOR WRITTEN CONSENT.

**Number of Subjects**	7 subjects: 4 T1DM patients and 3 healthy volunteers
**Inclusion Criteria**	**T1DM patients**<br>- Subjects willing to sign an informed consent form (ICF),<br>- Adult subjects, age ≥ 18, ≤ 50 years old<br>- Body Mass Index (BMI) 20≥, ≤ 27.5 [kg/m^2]<br>- Subjects willing to comply to study protocol requirements (exercises, ketone ester drinks, alcohol, confounders, study visits, blood sampling etc)<br>- Patients with type 1 diabetes mellitus (T1DM) according to WHO criteria, diagnosed for at least 12 months prior to screening<br>- Subjects being on insulin pump for at least 12 months<br>**Healthy Volunteers**<br>- Subjects willing to sign an informed consent form (ICF),<br>- Adult subjects, age ≥ 18, ≤ 50 years old<br>- BMI 20≥, ≤ 27.5<br>- Subjects willing to comply to study protocol requirements (exercises, ketone ester drinks, alcohol, study visits, blood sampling etc)<br>- Subject should be able to perform intense physical activity (exercise on bike); the investigator should – at this discretion – evaluate whether the participant is anticipated to be able to comply with this requirement<br>- Healthy subjects, as self-declared and confirmed by screening assessments and Principal Investigator's judgment<br>Note: Healthy status is defined by the absence of evidence of any active or chronic disease following a detailed medical and surgical history, a complete physical examination including vital signs, ECG, haematology, blood chemistry, serology, and urinalysis, as well as a high-level psychological assessment.
**Exclusion Criteria**	- Subjects with a contraindication to undergo challenging tests (i.e., ischemic heart disease, epilepsy, panhypopituitarism, hypoadrenalism, hypothyroidism, known allergic reaction to ibuprofen/paracetamol/acetylsalicylic acid)<br>- For people with diabetes: History of severe hypoglycaemia in the previous 6 months. Severe hypoglycaemia is defined as hypoglycaemia resulting in loss of consciousness or seizure<br>- For people with diabetes: History of diabetic ketoacidosis requiring emergency room visit or hospitalization in the previous 6 months<br>- Any blood disorder identified by haematocrit <30% or >55%<br>- History of hepatitis B, hepatitis C, or HIV<br>- A condition requiring or likely to require magnetic resonance imaging (MRI) during the study duration<br>- Female subjects who are pregnant, planning on becoming pregnant or nursing<br>- Any disorder, which in the investigator's opinion might jeopardise subject's safety or compliance with the protocol.<br>- Coagulation disorder, wound healing and bleeding disorder or taking anticoagulant medication<br>- Any long-term drug treatments other than insulin, such as statins, low-dose aspirin, fibrates etc.

CONFIDENTIAL - THIS DOCUMENT CONTAINS CONFIDENTIAL AND PROPRIETARY INFORMATION. THIS DOCUMENT MAY NOT BE REPRODUCED, USED, OR DISCLOSED TO ANY THIRD PARTY WITHOUT PRIOR WRITTEN CONSENT.

	- The presence of any other active implanted device except for insulin pumps (as defined further in protocol)<br>- The presence of any other CGM sensor or transmitter located in abdomen (other location is acceptable)<br>- Impaired fasting glucose or impaired glucose tolerance (for healthy volunteers)<br>- Any contraindication to the use of the YANG SYSTEM as listed in the device IFU (i.e. any known allergy to PDMS)
**Study Objectives**	**Primary Objectives:**<br>- Confirm safe integration of hardware in human subcutaneous tissue<br>- Confirm safety of surgical technique for implantation and explantation of YANG SENSOR<br>**Secondary Objective:**<br>- Assessment of 'easiness' of implantation/explantation procedure and investigation whether development of implantation tool is required to establish the safe and easy implantation/explantation surgical technique
**Endpoints**	**Primary Endpoints**<br>- Incidence of device-related or sensor insertion/removal procedure-related adverse events.<br>- Assessment of foreign body reaction due to subcutaneous implantation (i.e., inflammation, infection, tissue vascularization, formation of fibrotic scar tissue)<br>- Incidence of sensor failure (i.e., inability to collect the data).<br>**Secondary endpoints**<br>- Collection of users feedback in the form of clinical questionnaire to assess the 'easiness' of surgical procedure<br>- Requirements of duration of implantation and explantation procedure:<br>– Duration of pre-intervention preparation, intervention, and post-intervention phase (clean-up etc)<br>– Assessment of requirements for surgical tools and materials<br>– Personnel requirements: Surgeon time, nurse time, other personnel.<br>- Post explantation follow-up<br>**Exploratory endpoint:**<br>- Assessment of sensor ability to measure glucose and ketones and lactate levels for the development of the real-time algorithm<br>- Assessment of influence of interfering substances (i.e. ethanol, lactate, ketones, paracetamol, acetylsalicylic acid, sorbitol, fructose, aspartame, ibuprofen, caffeine and ascorbic acid [Vitamin C])
**Statistical Methodology**	No formal statistical analysis based on a predefined hypothesis will be performed for this early feasibility clinical investigation. The sample size is not statistically determined. Data will be presented using descriptive statistics, and the collected data regarding blood samples during measurement visits will serve only for the exploratory and safety purpose.

CONFIDENTIAL - THIS DOCUMENT CONTAINS CONFIDENTIAL AND PROPRIETARY INFORMATION. THIS DOCUMENT MAY NOT BE REPRODUCED, USED, OR DISCLOSED TO ANY THIRD PARTY WITHOUT PRIOR WRITTEN CONSENT.

**Analysis and Reporting**	The data regarding blood parameters collected during the measurement visits will serve only for the exploratory and safety purpose and will be used to develop the next version of YANG SENSOR. The data regarding related endpoints will be collected and analysed in order to confirm safe integration of hardware in human subcutaneous tissue and to confirm safety of surgical technique for implantation and explantation of YANG SENSOR.<br><br>All recorded and derived variables will be presented using appropriate descriptive summary statistics (continuous and ranked data: sample size, mean, standard deviation, minimum, first quartile, median, third quartile, maximum; categorical data: sample size, absolute and relative frequency).<br><br>All adverse events with onset during the study period will be displayed in summary tables. Tables will show the number of adverse events, the number and the percentage of patients affected by relation to implantation, explantation, device and underlying disease.<br><br>Accuracy for sensor glucose, ketones, and lactate will be reported as an exploratory endpoint in comparison with the blood reference values, using industry-standard accuracy metrics (MAD, MARD). The level of interference for ethanol, lactate, ketones, paracetamol, acetylsalicylic acid, sorbitol, fructose, aspartame, ibuprofen, caffeine and ascorbic acid [Vitamin C] will be reported in a quantitative and qualitative manner.<br><br>A final report will be generated after the last subject finished the study and after reviewing all data for correctness and plausibility. It will contain a description of the methodology and summary of the investigator's assessment of the medical device. The report will contain all data from all study participants in anonymous or pseudonymous form. No subject will be identified in the report or in the eventually published results.

CONFIDENTIAL - THIS DOCUMENT CONTAINS CONFIDENTIAL AND PROPRIETARY INFORMATION. THIS DOCUMENT MAY NOT BE REPRODUCED, USED, OR DISCLOSED TO ANY THIRD PARTY WITHOUT PRIOR WRITTEN CONSENT.

2 List of Abbreviations

ADE	Adverse Device Effect
AE	Adverse Event
BMI	Body Mass Index
CER	Clinical Evaluation Report
CGM	Continuous Glucose Monitoring
CIP	Clinical Investigation Plan
CRA	Clinical Research Associate
CRF	Case Report Form
CRO	Clinical Research Organization
DCCT	Diabetes Control and Complication Trial
DD	Device Deficiency
DKA	Diabetic Ketoacidosis
DMP	Data Management Plan
ECG	Electrocardiogram
eCRF	Electronic Case Report Form
EU	European Union
FAMHP	Federal Agency for Medicines and Health Products
FPFV	First Patient First Visit
HbA1c	Glycated Haemoglobin A1c
IB	Investigator Brochure
ICF	Informed Consent Form
isCGM	Intermittently Scanned CGM
ISF	Interstitial Fluid
ISO	International Organization for Standardization
LPLV	Last Patient Last Visit
MD	Medical Doctor
MDR	Medical Device Regulation
MRI	Magnetic resonance imaging
NIR	Near-Infrared
PIS	Patient Information Sheet
QoL	Quality of Life
rtCGM	Real Time CGM
SADE	Serious Device Effect
SAE	Serious Adverse Event
SMBG	Self-Monitoring of Blood Glucose
SOP	Standard Operating Procedure
T1DM	Type 1 Diabetes Mellitus
T2DM	Type 2 Diabetes Mellitus
UZA	Universitair Ziekenhuis Antwerpen (Antwerp University Hospital)
WHO	World Health Organization

CONFIDENTIAL - THIS DOCUMENT CONTAINS CONFIDENTIAL AND PROPRIETARY INFORMATION. THIS DOCUMENT MAY NOT BE REPRODUCED, USED, OR DISCLOSED TO ANY THIRD PARTY WITHOUT PRIOR WRITTEN CONSENT.

3 Investigational Device

3.1 Summary Description of the Investigational Device

For more detailed device description please refer to the corresponding section provided in the IB [PD-REP-00333 Investigator Brochure GLOW Study]

The YANG SYSTEM developed by Indigo Diabetes N.V. will be used for this early feasibility study on human subjects. The system is composed of YANG SENSOR (see Figure 1 (left)) and external device YANGEXD, that includes a data storage box and a wire-connected charging-antenna ('Donut' see Figure 1 (right))

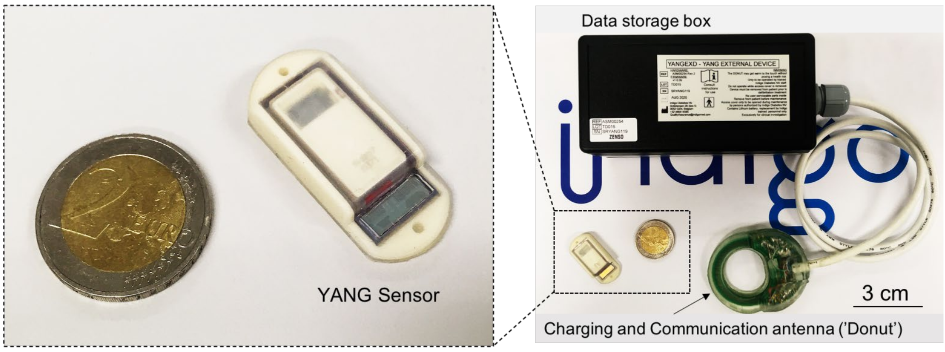

Figure 1 - YANG SENSOR (left) and YANGEXD (right), which includes a Storage Box and the 'Donut' antenna– The coin is for size reference only

The YANG SENSOR is an active implantable medical device as per AIMDD. It is intended to be implanted in the subcutaneous abdominal tissue, approximately 10mm below the skin. The SENSOR measures the glucose levels in the interstitial fluid by infrared absorption spectroscopy (in the 1550-2350 nm wavelength range). It is battery powered. The battery is wirelessly recharged via the 'Donut' of the YANGEXD that is applied to the skin using an adhesive patch at the level of the SENSOR. The YANG SENSOR wirelessly communicates its measurement data to the YANGEXD. YANG SENSOR is intended for single use. YANGEXD, the external device and remote control (which is part of the YANG SYSTEM) are intended to be reused.

The Indigo YANG SYSTEM is intended **exclusively** for clinical investigation. The system is intended to measure glucose, lactate and ketone levels only during planned study visits. The raw data will be collected by Indigo's team during the measurement visits and will not be used for the management of diabetes. The data will serve to develop the algorithm for the future version of the device.

3.2 Principle and Mechanism of Action

The YANG SYSTEM's mechanism of action relies on Near-Infrared (NIR) spectroscopy. The sensing principle is based on the direct interaction of light (in the form of an evanescent wave) with the medium (i.e. interstitial fluid), without needing intermediary chemistry such as electrochemical reactions. This sensing mechanism relies on the fact that each analyte absorbs specific light frequencies or wavelengths, i.e. each analyte has a signature spectral absorption curve. The shape of the absorption curve identifies the analyte. The amount of absorption indicates the concentration. The analytes of interest for diabetes management all respond in the near infrared (NIR) wavelength. The YANG SENSOR measures wavelengths in the 1550 to 2350 nm region, which covers the first overtone and the combination band of glucose, lactate and β-hydroxybutyrate. The YANG SENSOR is charged, and it wirelessly communicates its measurement data to the YANGEXD.

CONFIDENTIAL - THIS DOCUMENT CONTAINS CONFIDENTIAL AND PROPRIETARY INFORMATION. THIS DOCUMENT MAY NOT BE REPRODUCED, USED, OR DISCLOSED TO ANY THIRD PARTY WITHOUT PRIOR WRITTEN CONSENT.

3.3 Investigational Device Traceability and Accountability

Both parts of YANG SYSTEM (YANG SENSOR and YANGEDX) will have its unique serial number.

A device accountability system will be maintained during the study at the site; documenting device shipment and receipt, storage at the hospital, use, and return to the sponsor.

The YANG SYSTEM (YANG SENSOR and YANGEDX external device) accountability logs at the sponsor site will contain the following information:

- Device serial number
- Date of shipment
- Signature of person performing shipment
- Date of return*
- Signature of person receiving the return*
- Reason for return*

* will be completed upon device return, if applicable

The YANG SENSOR accountability log at the investigation site will contain the following information:

- Device serial number
- Date of reception
- Signature of person performing reception
- Date of use
- Implanted in: [patient identification]
- Date of implantation
- Date of explantation
- Not implanted: description (returned, discarded, etc.)
- Signature of person responsible for use

The YANGEDX accountability log at the investigation site will contain the following information:

- Device serial number
- Date of reception
- Signature of person performing reception
- Date of return*
- Signature of person receiving the return*
- Reason for return*

* will be completed upon device return, if applicable

With the signature, the investigator will acknowledge:

- The receipt of the clinical investigation supplies and agrees to use these devices only for patients who have given informed consent to this clinical investigation
- To store the devices correctly and in accordance with the specified requirements
- To keep records documenting the distribution of the devices to the patients.
- At the end of the study, the investigator will return all unused devices to the sponsor.

The device accountability form will contain all information for device identification (e.g. number of the device). This form will be completed and filed at sponsor's master file (original) and at the site in the investigator's site file (copy).

CONFIDENTIAL - THIS DOCUMENT CONTAINS CONFIDENTIAL AND PROPRIETARY INFORMATION. THIS DOCUMENT MAY NOT BE REPRODUCED, USED, OR DISCLOSED TO ANY THIRD PARTY WITHOUT PRIOR WRITTEN CONSENT.

4 Justification for the Design of the Clinical Investigation

4.1 Introduction

The ultimate goals of diabetes management include optimisation of glycaemic control to near-normoglycemia, minimising hypoglycaemia risk, prevention of diabetes-related complications and preserving quality of life (QoL)[1]. Research has shown that frequent blood glucose testing is the cornerstone of effective diabetes management. The Diabetes Control and Complication Trial (DCCT) (ClinicalTrials.gov Identifier: NCT00360815), one of the most important studies to date on the association between glucose control and long-term complications, found that regular glucose levels testing reduced the risk of developing long-term complications. Since there is currently no cure available for diabetes, self-management is the only solution to control the disease.

4.2 Investigational Device and Current Technology

Blood glucose self-monitoring with finger sticks (SMBG technique) has been the gold standard for glucose monitoring over decades. However, **real-time Continuous Glucose Monitoring (CGM)** has advanced at a fast pace since the first systems were introduced in 1999, and transformed the approach to diabetes care[2–6], including but not only because it can replace SMBG.

High cost and limited accessibility of CGM still today means many people with diabetes continue to rely on SMBG: however, this situation is changing since new CGM technologies become more approachable to the people with diabetes wordwide[7, 5]. The use of currently available CGM devices has significantly increased in western countries, especially for T1DM [7].

CGM technology provides a convenient, comprehensive assessment of blood glucose concentrations, allowing the identification of high and low glucose levels, in addition to evaluating glycaemic variability. CGM Systems can overcome many of the limitations of the clinical setting glycated haemoglobin A1c (HbA1c) based measurements while addressing the inconvenience and fragmented glucose data associated with self-monitoring of blood glucose (SMBG). CGM devices either continuously track the glucose concentration and provide near real-time data (rtCGM) or retrospectively show continuous measurements intermittently (isCGM) (i.e., when the user scans with the device). Intermittent devices include ''intermittently scanned" systems, from which stored data can be uploaded at any time [8].

The convenience of CGM systems relies on the fact that users have the opportunity to see not only what their glucose levels are at any given moment, but also how their glucose levels are changing over time. They can see instantaneously how nutrition, physical activity, and stress affect glucose levels. In addition, alerts and alarms of CGM systems provide the opportunity to catch and respond to hypoglycaemic events before glucose levels become dangerously low, improving safety of diabetes management [9].

Currently most commercially available CGM devices (i.e., Dexcom G6, Medtronic CGM, Abbott Freestyle LIBRE) use enzyme-based subcutaneous needle type sensors that measure interstitial glucose levels every 15-5 minutes [1]. The only commercially available subcutaneously implantable glucose sensor for rtCGM use is the Eversense system (manufactured by Senseonics, Maryland, USA) which was FDA-approved, and CE marked in 2017. Its measurement principle is based on optical fluorescence technology. The main disadvantage of Eversense is the invasive nature of sensor insertion requiring clinic attendance[1, 11]. However, there are many advantages of the implanted Eversense sensor compared to the needle-based subcutaneous sensors, such as the longer sensor life and slightly better accuracy. The sensor cannot be accidentally dislodged. It can be used in patients developing an allergic reaction to adhesives used in needle-based subcutaneous devices (often containing isobornyl acrylate). Moreover, individuals with cheiroarthropathy, low dexterity, that are contradicted to traditional CGM devices can be safely implanted with Eversense[11]. Last but not least, as reported by Barnard et al[12], fully implantable CGM Systems such as Eversense can bring psychological benefits to the users and improve their quality of life (QoL).

Indigo Diabetes N.V. is currently developing an active implantable medical device, intended to be used for real time, continuous measurement of glucose and ketone levels in the interstitial fluid in adults (18 years and older) with diabetes mellitus. The device will be intended for non-adjunctive use, to replace fingerstick blood glucose testing for taking

CONFIDENTIAL - THIS DOCUMENT CONTAINS CONFIDENTIAL AND PROPRIETARY INFORMATION. THIS DOCUMENT MAY NOT BE REPRODUCED, USED, OR DISCLOSED TO ANY THIRD PARTY WITHOUT PRIOR WRITTEN CONSENT.

diabetes treatment decisions. It aims to bring all clinical benefits as the discussed for the commercially available implantable glucose sensor. In addition, it will continuously monitor ketone levels and provide alerts and alarms to prevent diabetic ketoacidosis (DKA) events.

In this context, Indigo has developed a first prototype, named YANG SYSTEM, which will be used for this early feasibility study in humans.

YANG SYSTEM is an innovative system composed of the fully implantable YANG SENSOR and the external YANGEDX device. The System is not substantially similar to any other device currently available on the EU or US markets, since it combines continuous measurement of glucose with continuous β-hydroxybutyrate and lactate measurement. Its preliminary safety needs to be therefore assessed during this proof of concept feasibility study.

The objectives of GLOW study are to evaluate the integration of the YANG SENSOR's hardware in human subcutaneous tissue and to evaluate safety of surgical technique for SENSOR's implantation and explantation procedures. Clinical performance of the device will not be formally evaluated during this clinical investigation. The study is designed to enable data collection, which will be used to develop the software algorithm in a future version of the YANG SENSOR, in order to display measurements in real-time to the user.

5 Risk and Benefits of the Study Device and Investigation

5.1 Anticipated Clinical Benefits

The Indigo YANG SYSTEM is intended exclusively for clinical investigation. This clinical investigation is conduced as a part of the overall clinical development plan (see PD-PLN-530) for the CGM system, that Indigo aims to introduce to the market as a final device.

In the GLOW study, the device's purpose is data gathering for the calculation of glucose, ketones and lactate levels in the subcutaneous interstitial fluid of individuals with T1DM and healthy individuals. The raw data will be collected by Indigo's team during the measurement visits only and will not be used for the management of diabetes (for the subject group with diabetes). The study subjects will not be able to see the data generated by the SENSOR.

There are no clinical benefits anticipated for the GLOW study participants.

The study participants will be financially reimbursed for the participation in the trial (the expenses including transportation and time will be refunded in amount of 800 Euro per day for subjects in the "diabetes cohort" (up to 8000 Euro for completing all scheduled study visits) and 600 Euro per day for subjects in the "healthy subject cohort" (up to 6000Euro for completing all scheduled visits). The difference between the 2 groups is based on risk profile and differential intensity of the study tests/procedures.

The clinical benefit for the society and in particular for people with diabetes is related to the further development of next generation of YANG SENSOR, that will be available for all adults with T1DM and T2DM for the real-time management of their medical condition.

In this sense, the data collected during GLOW will allow to further develop a next-generation NIR-spectroscopy based sensor, capable of displaying glucose and ketone levels in real time. People with diabetes will be able to use this information to manage their medical condition, avoid hypoglycaemic events thanks to real-time alarms, and prevent potentially fatal episodes of diabetic ketoacidosis (DKA) due to real-time ketone alerts. Thus, the GLOW study is the first step taken by Indigo towards the development of CGM system that aims to improve diabetes control in line with clinical benefits associated to the current CGM devices, and prevent side effects of diabetes management, particularly related to DKA.

Thus, the additional intended benefit of Indigo's next generation device compared to benefits of currently available external, visible sensors is the positive psychological effect and improvement of users' QoL, and the ability to measure ketone levels in real-time, which is not possible with sensors currently available.

CONFIDENTIAL - THIS DOCUMENT CONTAINS CONFIDENTIAL AND PROPRIETARY INFORMATION. THIS DOCUMENT MAY NOT BE REPRODUCED, USED, OR DISCLOSED TO ANY THIRD PARTY WITHOUT PRIOR WRITTEN CONSENT.

5.2 Risks Associated with this Investigations and Residuals Risks

Indigo has conducted a Design Failure Mode and Effects Analysis (FMEA) focused on the risks inherent to the YANG SYSTEM design and clinical investigation procedures (implantation, explantation, measurement visits, collection of study endpoints). The results of the analysis demonstrate that YANG SYSTEM does not exhibit undesirable or intolerable risks to the study subjects nor to users and all risks associated with the use of investigational device are reduced as far as possible.

The anticipated AEs and device deficiencies (DDs) are described in section 7.5.2. For more detail refer to: [PD-REP-00333 Investigator Brochure GLOW Study]. The residual risks are also described with detail in IB (PD-REP-00333) as well as in current risk management report (see PD-REP-00622).

6 Study Objectives and Endpoints

6.1 Study Objectives

This is an open label, interventional, single-centre, prospective early feasibility study, designed to evaluate the initial device safety of the implanted device and the safety of the implant procedure while it will further enable data collection (that is raw NIR spectra of glucose, β-hydroxybutyrate and lactate levels from subjects' interstitial fluid), that will be used to develop the software algorithm to allow real-time measurements in a future version of the YANG SENSOR.

The study has two primary objectives:

- Confirm safe integration of hardware in human subcutaneous tissue
- Confirm safety of surgical technique for implantation and explantation of YANG SENSOR

The secondary objective is:

- Assessment of 'easiness' of implantation/explantation procedure and investigation whether development of implantation tool is required to establish the safe and easy implantation/explantation surgical technique

6.2 Study Endpoints and Variables

The GLOW study is an exploratory study; therefore, the clinical performance of the device will not be formally evaluated during this clinical investigation.

6.2.1 Primary Endpoints

In order to meet the primary objectives of the study (see Section 6.1) the following primary endpoints will be collected:

- Incidence of device-related or sensor insertion/removal procedure-related adverse events.
- Assessment of foreign body reaction due to subcutaneous implantation with biopsy (i.e., inflammation, tissue vascularization, formation of fibrotic scar tissue).
- Incidence of sensor failure (i.e., inability to collect the data).

6.2.2 Secondary Endpoints

Secondary endpoint is related to the confirmation of 'easiness' of implantation/explantation procedure

- Collection of users feedback in the form of clinical questionnaire to assess the 'easiness' of surgical procedure
- Requirements of duration of implantation and explantation procedure:
  - Duration of pre-intervention preparation, intervention, and post-intervention phase (clean-up etc)
  - Assessment of requirements for surgical tools and materials
  - Personnel requirements: surgeon time, nurse time, other personnel.

CONFIDENTIAL - THIS DOCUMENT CONTAINS CONFIDENTIAL AND PROPRIETARY INFORMATION. THIS DOCUMENT MAY NOT BE REPRODUCED, USED, OR DISCLOSED TO ANY THIRD PARTY WITHOUT PRIOR WRITTEN CONSENT.

- Post explantation follow-up

6.2.3 Exploratory Endpoints

- Assessment of sensor ability to measure glucose, β-hydroxybutyrate and lactate levels to allow the development of the algorithm
- Assessment of influence of interfering substances (i.e. ethanol, lactate, ketones, paracetamol, acetylsalicylic acid, sorbitol, fructose, aspartame, ibuprofen, caffeine and ascorbic acid [Vitamin C])

6.2.4 Collected Variables

The following variables will be collected during the study visits in the electronic CRF

- AE, SAE, SADE, DD, concomitant medication
- Demographic data (age, gender, ethnicity)
- Medical history data: general and diabetic (only for T1DM patients), including disease duration, treatment in the past (medication and interventions)
- Hypoglycaemia episodes assessment (only for T1DM patients) (i.e., timing of episodes, frequency and causes) at baseline
- Baseline physical examination data (height, weight, waist circumference, presence of lipohypertrophy, systolic and diastolic blood pressure determination)
- Baseline laboratory evaluation (haematology, blood chemistry, serology and urinalysis)
- Baseline assessment of general physical activity (questionnaire related to the frequency and intensity of sports)
- International physical activity questionnaire (IPAQ)
- Glucose, ketones, lactate levels measured in the blood samples:
  - Study subject 1/4: approx. 60 blood samples (0.5ml) will be withdrawn from the study participant per visit. The samples will be taken every 10 minutes in the hyperglycaemic range, and every 5 minutes in the hypoglycaemic range
  - Study subject 2: approx. 65 blood samples (0.5ml) will be withdrawn from the study participant per visit. The samples will be taken every 10 minutes in the hyperglycaemic range, every 5 minutes in the hypoglycaemic range and every 5 minutes during increasing and decreasing lactate levels, more specific starting when the lactate value has increased by 1 mM until it is back at 1 mM above the initial value
  - Study subject 3: approx. 70 blood samples (0.5 ml) will be withdrawn from the study participant per visit. The samples will be taken every 10 minutes in the hyperglycaemic range, every 5 minutes in the hypoglycaemic range and every 5 minutes during increasing and decreasing ketone levels, more specific starting when the ketone value has increased by 1 mM until it is back at 1 mM above the initial value.
  - Study subject 5-7: approx. 70 blood samples (0.5ml) will be withdrawn from the study participant per visit. The samples will be taken every 5 minutes during increasing and decreasing lactate/ketone levels, more specific starting when the lactate/ketone value has increased by 1 mM until it is back at 1 mM above the initial value, and every 10 minutes during the rest of the test.
  - **Note**: the exact number of samples depends on the time the subject spends in the certain range (hypoglycaemia range or increasing and decreasing lactate/ketones). The calculated numbers mentioned are an estimation and not a fixed number. The amount of fluid removed from the subject consists of 0,5 ml blood sample and 1 to 1,5 ml catheter waste liquid.
- Glucose levels measured by CGM (Dexcom G6) during study visits (Subjects 1-7)
- Heart rate values, measured by FitBit during study visits (all study subjects)
- Insulin delivery values during study visits (Note: T1DM patients only. The data will be downloaded from the participants' insulin pump)

CONFIDENTIAL - THIS DOCUMENT CONTAINS CONFIDENTIAL AND PROPRIETARY INFORMATION. THIS DOCUMENT MAY NOT BE REPRODUCED, USED, OR DISCLOSED TO ANY THIRD PARTY WITHOUT PRIOR WRITTEN CONSENT.

- Ethanol values measured with a breath analyser, every 10 minutes starting 30 minutes after the last alcohol consumption
- A biopsy of the encapsulation of the device taken in the subcutaneous tissue, during the explantation visit
- Prior to explant (maximum 5 days prior to explant) – subject to availability of an ultrasound device – ultrasound imaging may be performed to evaluate the depth of the sensor and the encapsulation (if any).
- Quantity and timing of oral glucose taken
- Requirements of duration of implantation and explantation procedure:
    - Duration of pre-intervention preparation, intervention, and post-intervention phase (clean-up etc)
    - Assessment of requirements for surgical tools and materials
    - Personnel requirements: Surgeon time, nurse time, other personnel.
    - Medical team and medical environment required for the procedure

7 Design of the Study

7.1 General

This is an open label, interventional, single-centre, prospective early feasibility study, designed to evaluate the initial device safety of the implanted device and the safety of the implant procedure while it will further enable data collection (that is raw NIR spectra of glucose, β hydroxy butyrate and lactate levels from subjects' interstitial fluid), that will be used to develop the software algorithm to allow real-time measurements in a future version of the YANG SENSOR.

Seven participants (4 T1DM patients and 3 healthy volunteers) will be enrolled. The study duration for every participant will be 28 days.

The subjects will participate in 6 measurement visits, one SENSOR implantation and one SENSOR explantation visit, as well as a follow-up visit approximately 10 days after SENSOR explant (wound evaluation and removal of sutures, if applicable) and a 15 minute phone call 4 weeks after the explantation (10 visits in total over the period of 57 days). During the measurement visits study participants will be subjected to glucose/lactate/ketone/ethanol challenging tests.

In addition, some study participants will be exposed to potential confounders of the glucose measurements: paracetamol, acetylsalicylic acid, sorbitol, fructose, aspartame, ibuprofen, caffeine and ascorbic acid [Vitamin C]. The YANG SENSOR will be turned on only during the 6 planned study measurement visits to collect the raw NIR spectra of glucose, ketones and lactate from the interstitial fluid of study participants. During the measurement visits, the subjects will be wearing the Fitbit device and commercially available CGM device (Dexcom G6). The SENSOR will be explanted at the 8th visit and upon explantation, a biopsy of the encapsulation of the device will be taken in the subcutaneous tissue.

CONFIDENTIAL - THIS DOCUMENT CONTAINS CONFIDENTIAL AND PROPRIETARY INFORMATION. THIS DOCUMENT MAY NOT BE REPRODUCED, USED, OR DISCLOSED TO ANY THIRD PARTY WITHOUT PRIOR WRITTEN CONSENT.

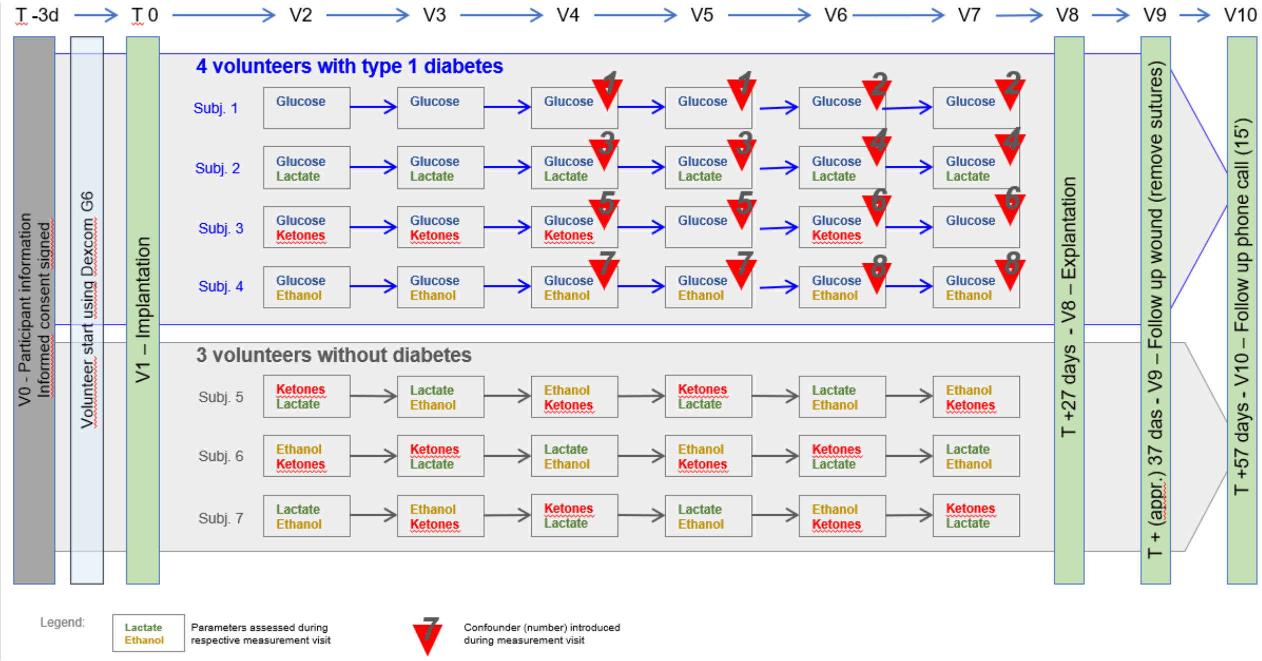

* The order of visits for the volunteers without diabetes can be changed to allow for study team organisational planning or at the request of the participant.

Figure 2 - Outline of the GLOW study visits

For more detail on study procedures refer to section 7.4.

7.2 Planned Study Steps

The following phases have been planned for this study:

1. Protocol and GCP training of site personnel prior to subjects' enrolment
2. Subjects enrolment with prior informed consent procedure
3. Implant and measurement visits and data acquisition
4. Data analysis and reporting

7.3 Test Subjects

Seven subjects will be enrolled in total to participate in the GLOW study. Four T1DM patients and three healthy volunteers.

7.3.1 Inclusion criteria

Adults with T1DM

- Subjects willing to sign an informed consent form (ICF),
- Adult subjects, age ≥ 18, ≤ 50 years old
- Body Mass Index (BMI) 20≥, ≤ 27.5 [kg/m2]
- Subjects willing to comply to study protocol requirements (exercises, ketone ester drinks, alcohol, study visits, blood sampling etc)

- Patients with type 1 diabetes mellitus (T1DM) according to WHO criteria, diagnosed for at least 12 months prior to screening
- Subjects being on insulin pump from at least 12 months

Adult Healthy Volunteers

- Subjects willing to sign an informed consent form (ICF),
- Adult subjects, age ≥ 18, ≤ 50 years old
- BMI 20≥, ≤ 27.5
- Subjects willing to comply to study protocol requirements (exercises, ketone ester drinks, alcohol, study visits, blood sampling etc)
- Subject should be able to perform intense physical activity (exercise on bike); the investigator should – at this discretion – evaluate whether the participant is anticipated to be able to comply with this requirement
- Healthy subjects, as self-declared and confirmed by screening assessments and Principal Investigator's judgment
  Note: Healthy status is defined by the absence of evidence of any active or chronic disease following a detailed medical and surgical history, a complete physical examination including vital signs, ECG, haematology, blood chemistry, serology and urinalysis, as well as a high-level psychological assessment

7.3.2 Exclusion Criteria

- Subjects with a contraindication to undergo challenging tests (i.e., ischemic heart disease, epilepsy, panhypopituitarism, hypoadrenalism, hypothyroidism, known allergic reaction to ibuprofen/paracetamol/acetylsalicylic acid)
- For people with diabetes: History of severe hypoglycaemia in the previous 6 months. Severe hypoglycaemia is defined as hypoglycaemia resulting in loss of consciousness or seizure
- For people with diabetes: History of diabetic ketoacidosis requiring room visit or hospitalization in the previous 6 months
- Any blood disorder identified by haematocrit <30% or >55%
- History of hepatitis B, hepatitis C, or HIV
- A condition requiring or likely to require magnetic resonance imaging (MRI) during the study duration
- Female subjects who are pregnant, planning on becoming pregnant or nursing
- Any disorder, which in the investigator's opinion might jeopardise subject's safety or compliance with the protocol.
- Coagulation disorder, wound healing and bleeding disorder or taking anticoagulant medication
- Any long-term drug treatments other than insulin, such as statins, low-dose aspirin, fibrates etc.
- The presence of any other active implanted device except for insulin pumps (as defined further in protocol)
- The presence of any other CGM sensor or transmitter located in abdomen (other location is acceptable)
- Impaired fasting glucose or impaired glucose tolerance (for healthy volunteers)
- Any contraindication to the use of the YANG SYSTEM as listed in the device IFU (i.e. any known allergy to PDMS)

7.3.3 Withdrawal Criteria

- Subjects willing to withdraw from the study. No special explanation required
- PI decision, based on the risk benefit assessment of the health status of the subject
- Subject becomes pregnant during the study
- Subject dropped out for adverse event or medical reasons

The subject can leave the investigation at any time, at the subject’s request. The reason for withdrawal will be investigated and carefully documented in the appropriate section of the Case Report Form, should the subject be willing

CONFIDENTIAL - THIS DOCUMENT CONTAINS CONFIDENTIAL AND PROPRIETARY INFORMATION. THIS DOCUMENT MAY NOT BE REPRODUCED, USED, OR DISCLOSED TO ANY THIRD PARTY WITHOUT PRIOR WRITTEN CONSENT.

to provide it. When a subject withdraws or is withdrawn from the study, the final evaluation will be performed as completely as possible. In addition, any comments (spontaneous or elicited) or complaints made by the subject or any other physician not related to the investigation, but taking care of the subject, will subsequently be carefully recorded in the relevant section of the Case Report Form.

7.3.4 Subjects Replacement

Study subjects which have completed 4 or more measurement visits will not be replaced. Study subjects which have completed less than 4 measurement visits will be replaced by a corresponding subject (i.e., T1DM subjects will be replaced by T1DM subjects; healthy volunteers will be replaced by healthy volunteers).

Identification and inclusion of the replacement subject is in the sole reasonability of the PI; Prof. Dr. med. Christophe De Block

7.3.5 Vulnerable Population

The clinical investigation does not aim to include any vulnerable population, nor it will be used in emergency procedures. If participants are screened for eligibility who might be considered belonging to a vulnerable population, appropriate measure are to be taken (e.g., employees will need to sign an additional consent form).

7.3.6 Informed Consent Process

The study subject will receive full oral and written information about the investigation using non-technical language and will be given time to decide whether to participate. The subject will then sign and date the written informed consent form (ICF) prior to entering into the investigation. The signed ICF will be maintained by the investigator in the clinical study file. A copy of the written information and of the signed consent form will be given to each subject.

The informed consent process must be conducted by an investigator or its designee. The person conducting the informed consent process will also sign and date the ICF.

In case important new information needs to be provided to the subject, he/she will be informed with a written information document. The document will have to be dated and signed by the subject to confirm he/she has understood the new information. The written information document must be approved by the Ethics Committees and the Competent Authority beforehand.

7.3.7 Subject Identification and Confidentiality

Subjects will be identified on all CRFs by a unique reference ID number. CRFs are confidential documents and will only be available to the sponsor (including sponsor delegates, like CRAs), the Investigator, and if requested to the Ethics Committee and Regulatory Authorities. The Principal Investigator will maintain a list identifying all subjects entered into the trial as part of the investigation file.

7.4 Procedures

7.4.1 Orientation and Training of Site Personnel Prior to Subject's Enrolment

All clinical staff involved in the study conduct will be trained during the study initiation visit by the Indigo representatives on the general GCP requirements and study protocol related procedures. This includes a training for the activities that need to be performed during the measurements (including how activities (i.e. standing bike) must be carried out). Prior to site activation, a specific training on device implantation will be provided to relevant study staff. Prior to site activation, the study staff will be trained on data collection (and data capture) and prior to be granted access to the electronic case report form system (eCRF), a training will be provided, and the study personnel will receive a strictly individual login and password.

7.4.2 Screening Procedures and Enrolment

Individuals with T1DM will be screened at the investigational site by the PI: Prof. Dr. med. Christophe De Block. Only the patients with confirmed T1DM, using an insulin pump for at least 12 months will be screened. The study information flyers will be distributed in the hospital to recruit the healthy volunteers, the contact information to the dedicated study coordinator will be provided on the flyer for the potential subjects willing to enter the study. All subjects will receive oral and written information and will be given enough time to consider their participation in the study. After signing the ICF, all subjects will be examined by the PI and checked for the eligibility criteria. Only after all inclusion and exclusion criteria are verified, subject will be considered enrolled and study procedures can be performed.

7.4.3 Baseline Physical Examination

The baseline evaluation (approx. 30-45 minutes) of a study participant will be conducted prior to the implantation procedure and will include the following:

1. Demographics including gender, age, ethnicity.
2. Medical history, general and diabetic (only for T1DM patients), including disease duration, treatment in the past (medication and interventions),
3. Baseline physical examination data (height, weight, waist circumference, presence of lipohypertrophy, systolic and diastolic blood pressure determination)
4. Baseline laboratory evaluation (haematology, blood chemistry, serology and urinalysis, pregnancy test)
5. Assessment of general physical activity (IPAQ questionnaire related to the frequency and intensity of sports)
6. Review of insulin pump settings (only T1DM patients)
7. Hypoglycaemia episodes assessment (only for T1DM patients) (i.e., timing of episodes, frequency and causes)

7.4.4 Implantation and Explantation Surgical Procedure

The YANG SENSOR will be implanted in the subcutaneous abdominal tissue, approximately 10mm below the skin. Implantation and explantation will be done using standard surgical technique to create a subcutaneous pocket. The surgery will be performed by the vascular surgeon from the investigational site, who will be trained by Indigo representative on surgical procedure.

Both implantation and explantation procedures will take approximately 90 minutes overall. All implantations and explantation will be done using local anaesthesia, without sedation. The first 3 devices will be implanted in a traditional operating room, while the remaining devices will be implanted in a treatment room typically used for out-patient ambulatory procedures.

A detailed description of procedure steps regarding the implant as well as the explant of the SENSOR, can be found in the IFU. Details on sampling of biopsy during the explant, is also provided in the IFU.

Please record during the procedure (both in- as explant) the duration of the procedure and the materials used to complete the in- or explant.

Prior to explant (by preference on the day of explant, but can be any day prior to explant, with a maximum of 5 days prior to explant) optional ultrasound imaging can be performed (based on the availability of an ultrasound device). The purpose of performing this imaging, is to confirm the subcutaneous implant depth of the sensor, and to evaluate the formation of encapsulation.

For investigation purposes (see also 7.4.7), it is required to take tissue sample biopsies during the explant procedures. These samples will be processed for further histological evaluation. In order this evaluation can be performed, the following steps must be followed during the explant procedure:

- After the incision is made to explant the device, the subcutaneous implant site should be visually inspected to identify the (early phase) encapsulation. Under direct visualisation, excise a tissue block (using a scalpel) of approximately 3x3 mm across the encapsulation from this “proximal site”.

CONFIDENTIAL - THIS DOCUMENT CONTAINS CONFIDENTIAL AND PROPRIETARY INFORMATION. THIS DOCUMENT MAY NOT BE REPRODUCED, USED, OR DISCLOSED TO ANY THIRD PARTY WITHOUT PRIOR WRITTEN CONSENT.

- Evaluate the possibility to perform the same excision at the distal part of the encapsulation after removal of the device. If this area is not accessible a punch biopsy of this distal area is also acceptable for histological analysis.
- All samples must be fixated immediately in a 3.5% formaldehyde solution and be made available to the concerned histological lab for further processing.

7.4.5 Post-Operative Management

After surgery, the postoperative management will be performed as per hospital standard of care wound management. All subjects will receive the 'Information sheet for post-operative precautions' and Implantation Card. This includes the guidance to avoid the intake of medication (including "over the counter") during the time the device is implanted and to refrain from intense activities that could inflict inappropriate strain on the implant/implant site.

Study subjects will be asked to monitor any sign of infection (i.e., redness, heat, pain, swelling) and will be asked to contact the study personnel in case of any inflammation symptoms to set up the appointment.

Following explantation, a follow-up to evaluate the explant site will be done after approximately 10 days following explant (this will be at the time of removal of suture material if applicable) and 30 days post-operatively (the latter by means of a phone call) (Visit 9 and Visit 10).

CONFIDENTIAL - THIS DOCUMENT CONTAINS CONFIDENTIAL AND PROPRIETARY INFORMATION. THIS DOCUMENT MAY NOT BE REPRODUCED, USED, OR DISCLOSED TO ANY THIRD PARTY WITHOUT PRIOR WRITTEN CONSENT.

7.4.6 Study Measurement Visits

The study participants will be the subjects of different challenging tests as presented on Figure 2 in Section 7.1:

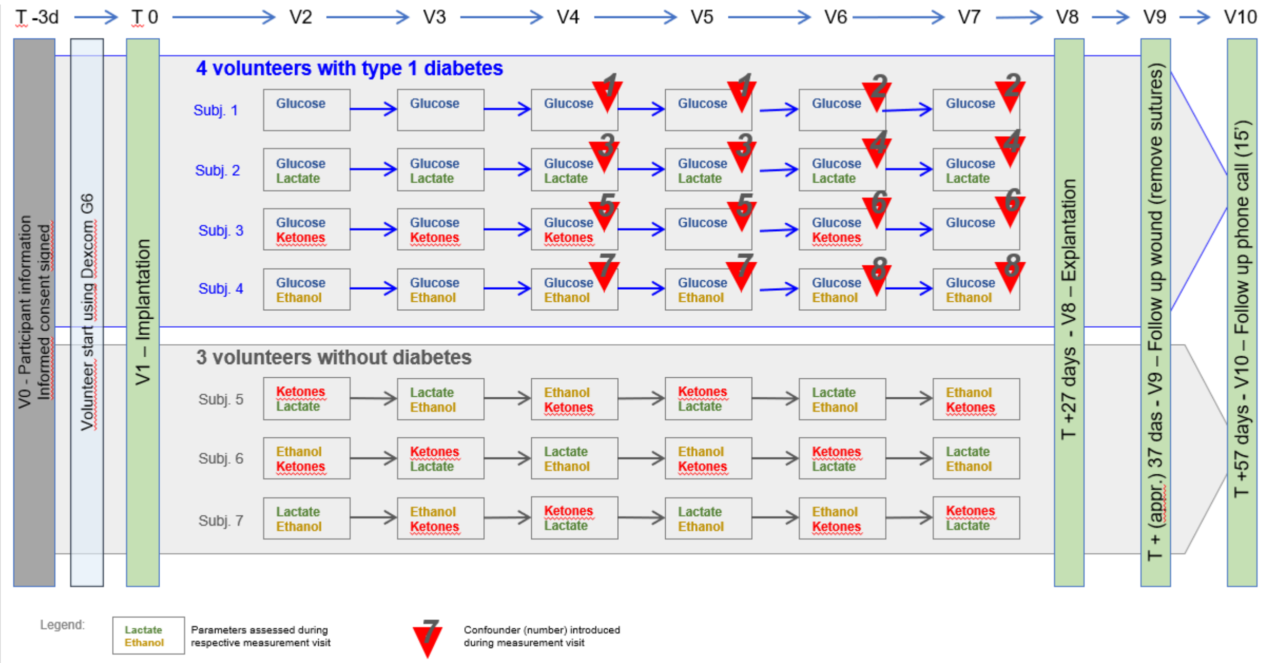

* The order of visits for the volunteers without diabetes can be changed to allow for study team organisational planning or at the request of the participant.

Figure 3 - Outline of the GLOW study visits

Each person with T1DM will be subjected to different challenging tests. This means, each subject will follow a different study protocol during the measurement visits.

4 different visit protocols are designed for T1DM patients as described in Table 1.

Measurement days 1 and 2 will establish a baseline. On measurement **days 3-6, a potential confounder will be added to the measurement day**. The confounder will be introduced when the subject's glucose levels are decreasing towards a hypoglycaemia at the moment the subject has a glucose value of approx. 300 mg/dl, single oral dose.

Each study subject 1-4 will be exposed to 2 different confounders, administered during 2 consecutive measurement days, according to the following table:

**Subject 1**	Measurement day 3+4: Confounder **1**	Measurement day 5+6 Confounder **2**
**Subject 2**	Measurement day 3+4: Confounder **3**	Measurement day 5+6: Confounder **4**
**Subject 3**	Measurement day 3+4: Confounder **5**	Measurement day 5+6: Confounder **6**
**Subject 4**	Measurement day 3+4: Confounder **7**	Measurement day 5+6: Confounder **8**

CONFIDENTIAL - THIS DOCUMENT CONTAINS CONFIDENTIAL AND PROPRIETARY INFORMATION. THIS DOCUMENT MAY NOT BE REPRODUCED, USED, OR DISCLOSED TO ANY THIRD PARTY WITHOUT PRIOR WRITTEN CONSENT.

The following confounders will be tested:

**Confounder 1**	Paracetamol (1000mg p.o.)	**Confounder 2**	Aspartame (2g p.o.)
**Confounder 3**	Acetylsalicylic acid (1000mg p.o.)	**Confounder 4**	Ibuprofen (1000 mg p.o.)
**Confounder 5**	Sorbitol (20g p.o.)	**Confounder 6**	Caffeine (4 espressos p.o)
**Confounder 7**	Fructose (50g p.o.)	**Confounder 8**	Vitamin C (2g p.o.)

**Study participant**	**Interferent**	**Protocol of measurement visits**	**Measured analyte**
T1DM patient<br>**Subject 1**	**Day 1-2** No interferent<br>**Day 3-4**: confounder 1<br>**Day 5-6** confounder 2	The visit will take around 8h in total (from 8am till 4pm).<br>The controlled hyperglycaemia and hypoglycaemia state will be induced in subject by controlling the insulin pump and glucose drink intake.<br>Glucose level will be monitored from starting point up to approx. 400mg/dl, then brought down to approx. 40mg/dl, and again up to normoglycemia. The max rate of change for decreasing glucose levels is 100mg/dl/hour.<br>The insulin pump will be stopped 1 hour prior to the examination. The subject will be asked to consume commercial glucose drink until his/her blood glucose level rises to approx. 400mg/dl. The insulin pump will be then turned on and the insulin will be given using personal insulin correction factor based upon the 1800 rule until blood glucose level drops to approx. 40mg/dl. After reaching that level the insulin pump will be switched off again and the subject will come back to the non-glycaemic level.<br>Approx. 60 blood samples (0.5ml each) will be withdrawn for the analysis by study nurse (sample withdrawal, when glucose level above 70mg/dl: every 10 minutes; below 70mgl/dl: every 5 minutes). Glucose levels will be additionally monitored by CGM device Dexcom G6.<br>Note: During the measurement visits on days 3-6; the confounders will be introduced when the subject's glucose levels are decreasing towards a hypoglycaemia at the moment the subject has a glucose value of approx. 300 mg/dl , single dose, per os.	Glucose only<br>Post hoc analysis of confounders 1 and 2
T1DM patient<br>**Subject 2**	**Day 1-2** Lactate<br>**Day 3-4**: lactate and confounder 3<br>**Day 5-6** lactate and confounder 4	The visit will take around 8h in total (from 8am till 4pm).<br>The controlled hyperglycaemia and hypoglycaemia state will be induced in subject by controlling the insulin pump.<br>Glucose level will be monitored from starting point up to approx. 250 mg/dl, then brought down to approx. 40mg/dl, and again up to normoglycemia. The max rate of change for decreasing glucose levels is 100mg/dl/hour.<br>The insulin pump will be stopped 1 hour prior to the examination. Blood glucose level will rise to approx. 250mg/dl. The insulin pump will then be turned on and the insulin will be injected using personal insulin correction factor based upon the 1800 rule until blood glucose level drops to approx. 40mgl/dl. After reaching that level the insulin pump will be switched off again and the subject will come back to the non-glycaemic level.<br>To measure the interference of lactate, subject 2 will be subjected to physical exercise, at least 1 hour after the start of the measurement visit once he/she has a CGM glucose value in the range of 150-180 mg/dl. Using a 'hometrainer' (ergometer) bicycle, the study subject will cycle for max. 2h with the goal of reaching a peak lactate level of 13mM. The subject will start with a build-up phase	Glucose and lactate<br>Post hoc analysis of confounders 3 and 4

CONFIDENTIAL - THIS DOCUMENT CONTAINS CONFIDENTIAL AND PROPRIETARY INFORMATION. THIS DOCUMENT MAY NOT BE REPRODUCED, USED, OR DISCLOSED TO ANY THIRD PARTY WITHOUT PRIOR WRITTEN CONSENT.

Study participant	Interferent	Protocol of measurement visits	Measured analyte
		of 30 minutes, in which the intensity goes up (linearly or stepwise). The subject needs to reach an intensity-plateau and hold that for half an hour. After this half hour, the subject can start the cooldown period, in which the intensity is decreased gradually for a period of 30 minutes. Lactate levels will be monitored continuously using blood samples.<br><br>Approx. 65 blood samples (0.5ml each) will be withdrawn for the analysis by study nurse (sample withdrawal, when glucose level above 70mg/dl: every 10 minutes; below 70mgl/dl: every 5 minutes and with increasing and decreasing lactate levels every 5 minutes, more specific starting when the lactate value has increased by 1 mM until it is back at 1 mM above the initial value). Glucose levels will be additionally monitored by CGM device Dexcom G6.<br><br>In addition, the Fitbit device will be used by subject2 to track physical activity. The data captured by Fitbit (heart rate, physical activity) are not captured by any other sensor and will allow quantification of the physical exercises during the respective measurement days.<br><br>Note: During the measurement visits on days 3-6; the confounders will be introduced when the subject's glucose levels are decreasing towards a hypoglycaemia at the moment the subject has a glucose value of approx. 185 mg/dl l , single dose, per os.	
T1DM patient<br><br>**Subject 3**	**Day 1**ketone and diet<br><br>**Day 2** ketone<br><br>**Day 3:** ketone, diet and confounder 5<br><br>**Day 4**: diet and confounder 5<br><br>**Day 5:** ketone and confounder 6<br><br>**Day 6** no interferent, only confounder 6	The visit will take around 8h in total (from 8am till 4pm).<br><br>The controlled hyperglycaemia and hypoglycaemia state will be induced in subject by controlling the insulin pump and glucose drink- intake.<br><br>Glucose level will be monitored from starting point up to approx. 400mg/dl, then brought down to approx. 40mg/dl, and again up to normoglycemia. The max rate of change for decreasing glucose levels is 100mg/dl/hour.<br><br>On each measurement day, the subject will be asked to consume commercial glucose drink until his/her blood glucose level rises to approx. 400mg/dl. The insulin pump will be then turned on and the insulin will be injected using personal insulin correction factor based upon the 1800 rule until blood glucose level drops to approx. 40mgl/dl. After reaching that level the insulin pump will be switched off again and the subject will come back to the non-glycaemic level.<br><br>**Day 1, 3 and 4:** to measure the interference of beta-Hydroxybutyrate study subject 3 will be requested to observe a low carb diet 3 days prior to measurement day 1, and throughout measurement days 3 and 4. On these days the study subject will stop his/her insulin pump 4 hours prior to the start of the measurement, with the goal of increasing endogenous ketone levels.<br><br>**Day 2, 5 and 6:** No low carb diet is required. The subject will only stop his/her insulin pump 1 hour prior to the start of the measurement.<br><br>**Day 1, 2, 3 and 5:** At the beginning of the measurements, ketone levels will be assessed. If inferior to 3.5mM, the study subject will receive a H.V.M.N. Ketone Ester drink in small increments, at least 1 hour after the start of the measurement visit. Initial dosing of the ketone ester will be half the recommended dose for athletes without diabetes, i.e. 115 mg/kg body weight. Subsequent increase of ketone ester shall not surpass a total intake of 330mg/kg body weight. Upon reaching a blood ketone concentration of 3.5mM, no additional ketone drink will be provided, and ketones will be allowed to return to normal levels spontaneously.	Glucose and ketone<br><br>Post hoc analysis of confounders 5 and 6

CONFIDENTIAL - THIS DOCUMENT CONTAINS CONFIDENTIAL AND PROPRIETARY INFORMATION. THIS DOCUMENT MAY NOT BE REPRODUCED, USED, OR DISCLOSED TO ANY THIRD PARTY WITHOUT PRIOR WRITTEN CONSENT.

Study participant	Interferent	Protocol of measurement visits	Measured analyte
		**Day 4 and 6:** no H.V.M.N Ketone Ester drink will be provided to the subject. On day 6 the subject will only be inducing controlled hyperglycaemia and hypoglycaemia state by controlling the insulin pump and glucose drink- intake.<br><br>During all measurement days, ketone levels will be monitored every 5 minutes.<br><br>Approx. 70 blood samples (2ml each) will be withdrawn for the analysis by study nurse (sample withdrawal, when glucose level above 70mg/dl: every 10 minutes; below 70mgl/dl: every 5 minutes, during increasing and decreasing ketone levels: every 5min, more specific starting when the ketone value has increased by 1 mM until it is back at 1 mM above the initial value). Glucose levels will be additionally monitored by CGM device Dexcom G6.<br><br>Note: During the measurement visits on days 3-6; the confounders will be introduced when the subject's glucose levels are decreasing towards a hypoglycaemia at the moment the subject has a glucose value of approx. 300 mg/dl , single dose, per os.	
T1DM patient<br><br>**Subject 4**	**Day 1** low ethanol<br><br>**Day 2** high ethanol<br><br>**Day 3:** low ethanol and confounder 7<br><br>**Day 4**: high ethanol and confounder 7<br><br>**Day 5:** low ethanol and confounder 8<br><br>**Day 6** high ethanol and confounder 8	The visit will take around 8h in total (from 8am till 4pm).<br><br>The controlled hyperglycaemia and hypoglycaemia state will be induced in subject by controlling the insulin pump and glucose drink intake.<br><br>Glucose level will be monitored from starting point up to approx. 400mg/dl, then brought down to approx. 40mg/dl, and again up to normoglycemia with rate of change: max. 100mg/dl/hour.<br><br>The insulin pump will be stopped, and the subject will be asked to consume glucose drink till his/her blood glucose level rises to approx. 400mg/dl. The insulin pump will be then turned on and the insulin will be injected until blood glucose level drops to approx. 40mg/dl. After reaching that level the insulin pump will be switched off again and the subject will come back to the non-glycaemic level.<br><br>To measure the interference of ethanol, subject 4 will be subjected to oral ethanol challenge, at least 1 hour after the start of the measurement visit; The aim is to have an increase of blood ethanol level of approx. 20mM (approx. 90 mg/dl). The increase of ethanol level will be achieved by the intake of alcohol-content beverages (strong beer or wine) by study subject. The subject will alternate a low ethanol day (around 3 high alcohol-content beverages) with a high alcohol day (max 6-8 high alcohol-content beverages), taking into account individual parameters that impact blood alcohol levels. Therefore, the numbers mentioned here are guiding, but can be adapted based on individual needs.<br><br>Approx. 60 blood samples (2ml each) will be withdrawn for the analysis by study nurse (sample withdrawal every 10 minutes). Glucose levels will be additionally monitored by CGM device Dexcom G6.<br><br>Note: During the measurement visits on days 3-6; the confounders will be introduced when the subject's glucose levels are decreasing towards a hypoglycaemia at the moment the subject has a glucose value of approx. 300 mg/dl , single dose, per os.	Glucose and ethanol<br><br>Post hoc analysis of confounders 7 and 8

Table 1 – Overview of study visits for T1DM patients

Dexcom G6 will be used by all study participants as additional comparator for glucose levels. Study participants will wear a continuous glucose sensor (CGM) during the experiments. The CGM measures glucose concentrations in the subcutaneous space, which shows fluctuations in glucose levels with a physiological delay to capillary measurements. Using the Dexcom G6 will allow quantification of this delay for the YANG SENSOR.

Note: the exact number of samples depends on the time the subject spends in the certain range (hypoglycaemia range or increasing and decreasing lactate/ketones). The calculated numbers mentioned are an estimation and not a fixed number. The amount of fluid removed from the subject consists of 0,5 ml blood sample and 1 to 1,5 ml catheter waste liquid.

Study protocol for the included three healthy volunteers differs from the protocol for people with T1DM. Two different interferents will be measured simultaneously during each study visit in each healthy volunteer. Every of three possible interferents combination (i.e., ketone/lactate; lactate/ethanol; ethanol/ketone) will be measured twice in every healthy subject (see Table 2).

**Study participant**	**Interferent combination**	**Protocol of measurement visits**	**Measured analyte**
Healthy volunteers<br>**Subject 5-7**	Ketones/Lactate	The visit will take around 8h in total (from 8am till 4pm).<br><br>Approx. 70 blood samples (0.5ml each) will be withdrawn for the analysis by study nurse (sample withdrawal during increasing and decreasing ketone or lactate levels; every 5 minutes, more specific starting when the lactate/ketone value has increased by 1 mM until it is back at 1 mM above the initial value). Glucose levels will be additionally monitored by CGM device Dexcom G6.<br><br>Study participants without diabetes will drink 60 ml H.V.M.N. Ketone Ester drink (which contains 30g ketones) at least 1 hour after the start of the measurement visit, in order to induce controlled ketoacidosis by increasing blood ketone level up to 5mM.<br><br>After at least 3 hours of ketone measurements, the study subject will exercise physically. Using a 'home trainer' (ergometer) bicycle, the study subject will cycle for max. 2h with the goal of reaching a peak lactate level of 13mM. The subject will start with a build-up phase of approx. 30 minutes, in which the intensity goes up (linearly or stepwise). The subject needs to reach an intensity-plateau and hold that for half an hour. After this half hour, the subject can start the cooldown period, in which the intensity is decreased gradually for a period of approx. 30 minutes. Lactate levels will be monitored continuously using blood samples.	Glucose ketone and lactate
	Ethanol/Ketones	The visit will take around 8h in total (from 8am till 4pm).<br><br>Approx. 70 blood samples (0.5ml each) will be withdrawn for the analysis by study nurse (sample withdrawal during increasing and decreasing ketones levels; every 5 minutes, more specific starting when the ketone value has increased by 1 mM until it is back at 1 mM above the initial value). Glucose levels will be additionally monitored by CGM device Dexcom G6.<br><br>To measure the interference of ethanol, participants will be subjected to oral ethanol challenge, at least 1 hour after the start of the measurement visit. The aim is to have an increase of blood ethanol level up to 20mM (approx. 90 mg/dl). The increase of ethanol levels will be achieved by the intake of 6-8 high alcohol-containing drinks (strong beer or wine) by study subject. After at least 3 hours of ethanol measurements, the study participants without diabetes will drink 60 ml H.V.M.N. Ketone Ester drink, which contains 30g ketones, in order to induce controlled ketoacidosis by increasing blood ketone level up to 5mM.	Glucose ketone and ethanol
	Lactate/Ethanol	The visit will take around 8h in total (from 8am till 4pm).<br><br>Approx. 70 blood samples (0.5ml each) will be withdrawn for the analysis by study nurse (sample withdrawal during increasing and decreasing lactate levels; every 5 minutes, more specific starting when the lactate value has	Glucose lactate and ethanol

CONFIDENTIAL - THIS DOCUMENT CONTAINS CONFIDENTIAL AND PROPRIETARY INFORMATION. THIS DOCUMENT MAY NOT BE REPRODUCED, USED, OR DISCLOSED TO ANY THIRD PARTY WITHOUT PRIOR WRITTEN CONSENT.

		increased by 1 mM until it is back at 1 mM above the initial value). Glucose levels will be additionally monitored by CGM device Dexcom G6.<br><br>To measure the interference of lactate, the study subject will be exercise physically, at least 1 hour after the start of the measurement visit. Using a 'home trainer' (ergometer) bicycle, the study subject will cycle intensively for max. 2h with the goal of reaching a peak lactate level of 13mM. The subject will start with a build-up phase of approx. 30 minutes, in which the intensity goes up (linearly or stepwise). The subject needs to reach an intensity-plateau and hold that for half an hour. After this half hour, the subject can start the cooldown period, in which the intensity is decreased gradually for a period of approx. 30 minutes. Lactate levels will be monitored continuously using blood samples.<br><br>After at least two hours of lactate measurements participants will be subjected to oral ethanol challenge. The aim is to have an increase of blood ethanol level up to 20mM (approx. 90 mg/dl). The increase of ethanol levels will be achieved by the intake of 3-4 high alcohol-containing drinks (strong beer or wine) by study subject. These numbers can be considered as guidance since individual response to alcohol intake is subject to important inter-individual differences.	

Table 2 - Overview of study visits for healthy volunteers

7.4.7 Investigational Device Explantation

YANG SENSOR will be explanted from the abdominal tissue of study subject during the 8th study visit by the vascular surgeon from the investigational site. A biopsy of the encapsulation of the device with a 3 mm margin will be taken of the subcutaneous tissue to evaluate sensor integrity in subcutaneous tissue and to confirm the implantation safety.

Obtain tissue biopsy samples according to the following steps: After the incision is made to explant the device, the subcutaneous implant site should be visually inspected to identify the (early phase) encapsulation. Under direct visualisation, excise a tissue block (using a scalpel) of approximately 3x3 mm across the encapsulation from this "proximal site".

- Evaluate the possibility to perform the same excision at the distal part of the encapsulation after removal of the device. If this area is not accessible a punch biopsy of this distal area is also acceptable for histological analysis.
- All samples must be fixated immediately in a 3.5% formaldehyde solution and be made available to the concerned histological lab for further processing.

Explanted devices will be disinfected by immersion in a liquid disinfectant, sealed in a plastic bag that is marked 'BioHazard'. The sponsor will then collect all the devices at the hospital.

Note: All the devices that will require explantation prior to subjects' termination of study procedures (i.e., due to tissue reaction, patient incompatibility, sensor failure etc.) will be collected by the sponsor from the hospital, the reason of explantation will be explained in Case Report Form and all the cases will be carefully investigated.

7.4.8 Follow-up

Following explantation, the study subjects will have a follow-up visit approximately 10 days post explant (to coincide with removal of sutures if applicable) and 4 weeks post-explantation, the latter by phone call.

7.4.9 Summary of Study Activities

The following table provides an overview of the planned procedure steps.

CONFIDENTIAL - THIS DOCUMENT CONTAINS CONFIDENTIAL AND PROPRIETARY INFORMATION. THIS DOCUMENT MAY NOT BE REPRODUCED, USED, OR DISCLOSED TO ANY THIRD PARTY WITHOUT PRIOR WRITTEN CONSENT.

	**Study Periods**							
	**Patient Information**	**Screening Visit V0**	**Implantation Visit V1**	**Measurement Visits V2- V3**	**Measurement Visits V4- V7**	**Explantation Visit V8**	**Post explant visit V9**	**Final visit (phone call) V10**
Time related to subject implantation (days)	**>-14**	**-14**	**0**	**1-28**		**28**	**Appr 37**	**57**
Patient Information	X							
Signing of Informed Consent Form		X						
Demographics		X						
Medical History, incl. specific diseases		X						
Eligibility Criteria		X						
Physical Examination		X				X		
Wound evaluation				X	X	X	X	
Vital Signs		X	X			X		
Laboratory Tests		X						
Patient Inclusion		X						
CGMS mounting and instruction			X					
User instruction for FitBit and CGM as required			X					
Glucose, ketones, lactate levels measured in the blood samples				X*	X*			
Glucose levels measured by CGM (Dexcom G6)				X*	X*			
Confounder application					X*			
HR values, measured by FitBit				X*	X*			
Insulin delivery values registration				X*	X*			
Quantity and timing of oral glucose taken				X*	X*			
Ethanol values measured with a breath analyser				X*	X*			
Evaluation of abdominal skin condition			X					
SENSOR implantation			X					
SENSOR explantation						X		
A biopsy of the encapsulation of the device taken in the subcutaneous tissue						X		
Recording of AE, SAE, SADE, DD, and concomitant medication			X	X	X	X	X	X
Ultrasound imaging of sensor implantation site (optional)						X (max 5 days prior to explant)		

* the measured variable will depend on study procedures associated to each study subject

Table 3 - Summary of Study Activities

CONFIDENTIAL - THIS DOCUMENT CONTAINS CONFIDENTIAL AND PROPRIETARY INFORMATION. THIS DOCUMENT MAY NOT BE REPRODUCED, USED, OR DISCLOSED TO ANY THIRD PARTY WITHOUT PRIOR WRITTEN CONSENT.

7.5 Adverse Events, Adverse Device Effects and Device Deficiencies

For this clinical investigation, the definitions of ISO 14155: 2020 and of MEDDEV 2.7/3 guidance will be applied.

7.5.1 Definitions

Adverse Event (AE): - Any untoward medical occurrence, unintended disease or injury, or untoward clinical signs (including abnormal laboratory findings) in subjects, users or other persons, whether or not related to the investigational medical device.

This definition includes events related to the investigational medical device or the comparator and events related to the procedures involved. For users or other persons, this definition is restricted to events related to investigational medical device.

Serious Adverse Event (SAE): - Adverse event that led to any of the following

a) death,
b) serious deterioration in the health of the subject, users or other persons as defined by one or more of the following:

- a life-threatening illness or injury, or
- a permanent impairment of a body structure or a body function including chronic diseases, or
- in-patient or prolonged hospitalization, or
- medical or surgical intervention to prevent life-threatening illness or injury or permanent impairment to a body structure or a body function,

c) foetal distress, foetal death or a congenital abnormality or birth defect including physical or mental impairment

Adverse Device Effect (ADE): - Adverse event related to the use of an investigational medical device

This definition includes adverse events resulting from insufficient or inadequate instructions for use, deployment, implantation, installation, or operation, or any malfunction of the investigational medical device. It also includes include any event resulting from use error or from intentional misuse of the investigational medical device.

Serious Adverse Device Effect (SADE): - Adverse device effect that has resulted in any of the consequences characteristic of a serious adverse event.

Device Deficiency (DD): - Inadequacy of a medical device with respect to its identity, quality, durability, reliability, usability, safety or performance. Device deficiencies include malfunctions, use errors, and Inadequacy in the information supplied by the manufacturer including labelling. This definition also includes device deficiencies related to the investigational medical device or the comparator.

Severity of Adverse Events/Adverse Device Effects

The severity of clinical AEs /ADEs is graded on a three-point scale: mild, moderate, severe, and reported on specific AE pages of the CRF. If the severity of an AE/ADE worsens during medical device administration, only the worst intensity should be reported on the AE page. If the AE lessens in intensity, no change in the severity is required.

Mild

Event may be noticeable to subject; does not influence daily activities; the AE /ADE resolves spontaneously or may require minimal therapeutic intervention;

Moderate

Event may make subject uncomfortable; performance of daily activities may be influenced; intervention may be needed; the AE/ADE produces no sequelae.

Severe

CONFIDENTIAL - THIS DOCUMENT CONTAINS CONFIDENTIAL AND PROPRIETARY INFORMATION. THIS DOCUMENT MAY NOT BE REPRODUCED, USED, OR DISCLOSED TO ANY THIRD PARTY WITHOUT PRIOR WRITTEN CONSENT.

Event may cause noticeable discomfort; usually interferes with daily activities; subject may not be able to continue in the study; the AE/ADE produces sequelae, which require prolonged therapeutic intervention.

Relationship to medical device

For the purpose of harmonizing reports, each SAE will be classified according to five different levels of causality. The sponsor and the investigators will use the following definitions to assess the relationship of the serious adverse event to the investigational medical device or procedures:

1) **Not related**: relationship to the device or procedures can be excluded when:

- the event is not a known side effect of the product category the device belongs to or of similar devices and procedures;
- the event has no temporal relationship with the use of the investigational device or the procedures;
- the serious event does not follow a known response pattern to the medical device (if the response pattern is previously known) and is biologically implausible;
- the discontinuation of medical device application or the reduction of the level of activation/exposure - when clinically feasible - and reintroduction of its use (or increase of the level of activation/exposure), do not impact on the serious event;
- the event involves a body-site or an organ not expected to be affected by the device or procedure;
- the serious event can be attributed to another cause (e.g. an underlying or concurrent illness/ clinical condition, an effect of another device, drug, treatment or other risk factors);
- the event does not depend on a false result given by the investigational device used for diagnosis, when applicable;
- harms to the subject are not clearly due to use error;
- In order to establish the non-relatedness, not all the criteria listed above might be met at the same time, depending on the type of device/procedures and the serious event.

2) **Unlikely**: the relationship with the use of the device seems not relevant and/or the event can be reasonably explained by another cause, but additional information may be obtained.

3) **Possible:** the relationship with the use of the investigational device is weak but cannot be ruled out completely. Alternative causes are also possible (e.g. an underlying or concurrent illness/ clinical condition or/and an effect of another device, drug or treatment). Cases where relatedness cannot be assessed, or no information has been obtained should also be classified as possible.

4) **Probable:** the relationship with the use of the investigational device seems relevant and/or the event cannot reasonably be explained by another cause, but additional information may be obtained.

5) **Causal relationship**: the serious event is associated with the investigational device or with procedures beyond reasonable doubt when:

- the event is a known side effect of the product category the device belongs to or of similar devices and procedures;
- the event has a temporal relationship with investigational device use/application or procedures;
- the event involves a body-site or organ that
  - the investigational device or procedures are applied to;
  - the investigational device or procedures have an effect on;
- the serious event follows a known response pattern to the medical device (if the response pattern is previously known);

CONFIDENTIAL - THIS DOCUMENT CONTAINS CONFIDENTIAL AND PROPRIETARY INFORMATION. THIS DOCUMENT MAY NOT BE REPRODUCED, USED, OR DISCLOSED TO ANY THIRD PARTY WITHOUT PRIOR WRITTEN CONSENT.

- the discontinuation of medical device application (or reduction of the level of activation/exposure) and reintroduction of its use (or increase of the level of activation/exposure), impact on the serious event (when clinically feasible);
- other possible causes (e.g. an underlying or concurrent illness/ clinical condition or/and an effect of another device, drug or treatment) have been adequately ruled out;
- harm to the subject is due to error in use;
- the event depends on a false result given by the investigational device used for diagnosis 17, when applicable;
- In order to establish the relatedness, not all the criteria listed above might be met at the same time, depending on the type of device/procedures and the serious event.

Unanticipated serious adverse device effect (USADE):

Serious adverse device effect which by its nature, incidence, severity or outcome has not been identified in the current version of the risk analysis report. Anticipated serious adverse device effect (ASADE) is an effect which by its nature, incidence, severity or outcome has been identified in the risk analysis report.

7.5.2 Anticipated Adverse Events and Device Deficiencies

This section lists the possible adverse events, adverse device effects and their definition. Included in the list are all known adverse events, even if they can't be directly attributed to the use of the Investigational device.

The list of expected adverse events, adverse device effects and device deficiencies was divided according to the severity and probability grades. For the detail information please refer to [reference to FMEA]

The following shall be considered as anticipated Adverse Events (AEs) in the specific scope of this clinical investigation (see Table 4).

Note: According to the risk analysis performed by Indigo, only the AEs classified as mild have higher probability of occurrence. Due to implementation of appropriate mitigation measures, all the moderate and severe AEs listed in the table below although probable, are very unlikely to occur during the planned clinical investigation.

**AEs related to implantation procedures**		
**Mild**	**Moderate**	**Severe**
- Pain and discomfort,<br>- Irritation, redness on implantation site<br>- Bleeding	- Prolonged surgical procedure<br>- Infection at the implantation site<br>- Eczema<br>- Moderate allergic reaction<br>- Implant require explantation and/or replacement<br>- Moderate wound healing complication due to diabetes (only T1DM patients)	- Severe wound healing complication due to diabetes (only T1DM patients)<br>- Severe allergic reaction
**AEs related to study procedures (measurement visits)**<br>**DKA, Hyper and hypoglycaemia events (glucose and ketones challenging)**		
**Mild**	**Moderate**	**Severe**
- Blurred or Double Vision<br>- Thirst<br>- Fatigue<br>- Headache	- Nausea and vomiting<br>- Shakiness, Clumsiness or Jerky Movements<br>- Shortness of breath<br>- Abdominal pain	- Loss of consciousness<br>- Coma<br>- Death

CONFIDENTIAL - THIS DOCUMENT CONTAINS CONFIDENTIAL AND PROPRIETARY INFORMATION. THIS DOCUMENT MAY NOT BE REPRODUCED, USED, OR DISCLOSED TO ANY THIRD PARTY WITHOUT PRIOR WRITTEN CONSENT.

- Weakness<br>- Hunger<br>- Dizziness<br>- Sweating<br>- Fast Heartbeat<br>- Confusion<br>- Irritability<br>- Anxiety<br>- Drowsiness		
**AEs related to study procedures (measurement visits)**<br><br>**Paracetamol intake during glucose challenging**		
**Mild**	**Moderate**	**Severe**
NA	- Nausea and Vomiting<br>- Abdominal Pain<br>- Diarrhoea<br>- Allergic reaction (i.e., mild symptoms of rush and itching, swelling of the face and throat, runny nose, difficulty in breathing)<br>- Headache or dizziness	- Liver failure<br>- Severe allergic reaction (i.e., severe symptoms of rush and itching, swelling of the face and throat, runny nose, difficulty in breathing)
**AEs related to study procedures (measurement visits)**<br><br>**Aspartame intake during glucose challenging**		
**Mild**	**Moderate**	**Severe**
- Headache	NA	NA
**AEs related to study procedures (measurement visits)**<br><br>**Acetylsalicylic acid or ibuprofen intake during glucose and lactate challenging**		
**Mild**	**Moderate**	**Severe**
-	- Allergic reaction (i.e., mild symptoms of rush and itching, swelling of the face and throat, runny nose, difficulty in breathing)<br>- Nausea and Vomiting<br>- Abdominal Pain<br>- Diarrhoea<br>Increased tendency for bleeding	- Severe allergic reaction (i.e., severe symptoms of rush and itching, swelling of the face and throat, runny nose, difficulty in breathing)<br>- Unusual bleeding (i.e., coughing up blood, blood in your vomit or urine, or black stools)
**AEs related to study procedures (measurement visits)**<br><br>**Sorbitol intake during glucose and ketone challenging**		
**Mild**	**Moderate**	**Severe**
- gastrointestinal disturbances (i.e. bloating, abdominal discomforts)	- Diarrhoea	NA
**AEs related to study procedures (measurement visits)**<br><br>**Caffeine intake during glucose and ketone challenging**		
**Mild**	**Moderate**	**Severe**
- Headache	- Increased heart rate<br>- Fatigue	- NA
**AEs related to study procedures (measurement visits)**<br><br>**Vitamin C intake during glucose and ethanol challenging**		
**Mild**	**Moderate**	**Severe**

CONFIDENTIAL - THIS DOCUMENT CONTAINS CONFIDENTIAL AND PROPRIETARY INFORMATION. THIS DOCUMENT MAY NOT BE REPRODUCED, USED, OR DISCLOSED TO ANY THIRD PARTY WITHOUT PRIOR WRITTEN CONSENT.

- Nausea and Vomiting	- Nausea and Vomiting<br>- Diarrhoea<br>- Heartburn	- Kidney stones
**AEs related to study procedures (measurement visits)**<br>**Physical activity (lactate challenging)**		
**Mild**	**Moderate**	**Severe**
- Neck pain<br>- Low back pain<br>- Muscle pain	- Saddle sores; caused by skin breakdown from pressure and friction (ranged in severity from mild chafing to ulcers)<br>- Handlebar palsy; typically include numbness and tingling in the fifth digit and the half of the ring finger adjacent to the fifth digit<br>- Patellofemoral pain syndrome (PFPS), or cyclist's knee, is an overuse condition caused by repetitive friction between the kneecap and the thigh bone<br>- Burning and tingling in the feet (metatarsalgia, "hot foot" syndrome, foot numbness)<br>- Dehydration	NA
**AEs related to study procedures (measurement visits)**<br>**Alcohol consumption (ethanol challenging)**		
**Mild**	**Moderate**	**Severe**
- Increased blood pressure<br>- Dizziness<br>- Increased hart rate<br>- Hangover (worsens when mixing ketones with alcohol)<br>- Lowered self-esteem	- Nausea and vomiting	NA
**AEs related to study procedures (measurement visits)**<br>**Ketone rich beverage consumption (ketone challenging)**		
**Mild**	**Moderate**	**Severe**
- Stomach Upset<br>- Shakiness<br>- Dizziness<br>- Sweating<br>- Hunger<br>- Fast Heartbeat<br>- Confusion<br>- Irritability<br>- Anxiety<br>- Headache,<br>- Blurry or Double Vision,<br>- Drowsiness,	- Dehydration<br>- Shakiness, Clumsiness or Jerky Movements	- Loss of consciousness<br>- Death

CONFIDENTIAL - THIS DOCUMENT CONTAINS CONFIDENTIAL AND PROPRIETARY INFORMATION. THIS DOCUMENT MAY NOT BE REPRODUCED, USED, OR DISCLOSED TO ANY THIRD PARTY WITHOUT PRIOR WRITTEN CONSENT.

AEs related to explantation procedures		
**Mild**	**Moderate**	**Severe**
- Pain/discomfort<br>- Irritation, redness<br>- Eczema<br>- Bleeding	- Prolonged surgical procedure<br>- Infection at the implantation site<br>- Moderate wound healing complication due to diabetes (only T1DM patients)	- Severe wound healing complication due to diabetes (only T1DM patients)
**AEs related to collection of endpoints (blood sampling)**		
**Mild**	**Moderate**	**Severe**
- Pain/discomfort at the sampling site,<br>- Bruising,<br>- Need of additional sampling, repetition of study procedures	- Haemolysis<br>- Haematoma and injury to anatomical structures in the vicinity of the needle entry<br>- Infection, eczema, need for antibiotic medication	NA
**AEs related to collection of endpoints (glucose measurements by Dexcom G6)**		
**Mild**	**Moderate**	**Severe**
- Skin reaction at the insertion site; i.e., hematoma: bruising, erythema, pain/discomfort	- Moderate allergic reaction,<br>- Infection due to contamination	- Severe allergic reaction
**AEs related to collection of endpoints (biopsy)**		
**Mild**	**Moderate**	**Severe**
- Pain/discomfort	- Bleeding<br>- Moderate healing problems due to diabetes complication (T1DM patients only)	- Severe healing problems due to diabetes complication (people with T1DM only)
**AEs related to device failures and deficiencies**		
**Mild**	**Moderate**	**Severe**
- Prolonged surgical procedure	- Temperature induced tissue damage<br>- Systemic allergic reaction<br>- Implant may need to be surgically removed, adjusted or replaced	- Severe subcutaneous foreign body reaction (fibrotic tissue)

Table 4 - Anticipated adverse events, according to its severity

Anticipated Device Deficiencies (DDs) according to its severity (see Table 5).

DDs related to YANG SENSOR		
**Mild**	**Moderate**	**Severe**
- NA	- Sensor overheating<br>- Sensor breakage<br>- Breach of hermeticity of encapsulation<br>- Sensor membrane crack/clogging<br>- Migration of the Sensor	- Device not properly sterilized<br>- Device failure due to encapsulation<br>- Battery electrolyte leakage<br>- Sensing material leakage

CONFIDENTIAL - THIS DOCUMENT CONTAINS CONFIDENTIAL AND PROPRIETARY INFORMATION. THIS DOCUMENT MAY NOT BE REPRODUCED, USED, OR DISCLOSED TO ANY THIRD PARTY WITHOUT PRIOR WRITTEN CONSENT.

	- Pieces of fractured material in the surrounding tissue	
**DDs related to YANGEDX**		
**Mild**	**Moderate**	**Severe**
- NA	- NA	- NA

Table 5 - Anticipated device deficiencies, according to its severity

According to FMEA performed by Indigo (QA-REP-00464_v0.4_GLOW_SYSTEM_FMEA), all the risks leading to potential device deficiencies, listed in Table 5 are reduced as much as possible due to the implemented mitigation measures (i.e., V&V activities).

Moreover, as stated in the current risk analysis and in line with the state-of-the-art clinical literature (see PD-REP-00152 – Clinical Evaluation Report and PD-REP-00333 Investigator Brochure) only the risks of DDs classified as ‘*sensor breakage*’, ‘*sensor migration*’ and ‘*communication disturbance*’ have higher probability of occurrence. All the other risks of DDs listed in Table 5 are very unlikely to occur during the planned clinical investigation and should be considered as negligible.

7.5.3 Adverse Events Recording Procedure

The eCRF prepared for the study contains an Adverse Event report form that follows the guidance provided by the MEDDEV 2.7/3. One form per Event must be used. The following events must be recorded using this form:

- Any Adverse Event – Considering the duration of the investigation per patient, any Adverse Event shall be recorded.
- Any Investigational Medical Device Deficiency
- New findings/updates in relation to already reported events.

7.5.4 Adverse Events Reporting Procedure

For this clinical investigation, the rules set out in EU 2017/745, ISO 14155: 2020 and in MEDDEV 2.7/3 guidance will be applied. All serious adverse events and all serious adverse device effect which are either possibly or definitively related to the SYSTEM or to the intervention will be reported.

According to The Federal Agency for Medicines and Health Products of Belgium (FAMHP) website: *“All serious adverse events must be fully recorded and immediately notified to all competent authorities of the Member States in which the clinical investigation is being performed.*

All those serious adverse events must be reported to ct.rd@fagg.be(link sends e-mail) by using the European form(This hyperlink opens a new window)“.

CONFIDENTIAL - THIS DOCUMENT CONTAINS CONFIDENTIAL AND PROPRIETARY INFORMATION. THIS DOCUMENT MAY NOT BE REPRODUCED, USED, OR DISCLOSED TO ANY THIRD PARTY WITHOUT PRIOR WRITTEN CONSENT.

Reporting - from Investigator to Sponsor:

The investigator must report all any SAE, device related or not, and irrespective of the potential causal relationship to the study to the sponsor immediately but not later than within 3 calendar days after the investigator has become aware of the event preferably by completing an Adverse Event form within the eCRF In case of death or SAEs requiring urgent medical interventions, the event must be reported within 24 hours of the investigator becoming aware of the SAE. Non-serious AEs are to be reported as soon as possible.
In case the eCRF is not available, the reporting may be performed by announcing the event to Indigo's Chief Medical Officer **Dr. med. Gijs Klarenbeek** by phone at + 32 479 98 64 58 or by sending a copy of the completed adverse event form by email to gijs.klarenbeek@indigomed.com and SAE@clinfidence.com.

Reporting - from Sponsor to Competent Authority

The sponsor must report all reportable events to the Competent Authority immediately but not later than within 7 calendar days after the receipt of the report of the event from the investigator.

Reporting - from Investigator to Ethics Committee

The investigator must report all reportable events to his Ethics Committee immediately after reception of the Adverse Event report form from the sponsor but not later than within 3 calendar days after reception of the form.

During each visit, all adverse events, whether voluntarily reported by the patient or observed by the investigators, will be documented in the appropriate forms as described in sections 7.5.4.1 and 7.5.4.2.

7.5.4.1 Adverse Event Form

The adverse event form will contain the following information:

- Subject identification
- Date of adverse event
- Description of adverse event
- Determination of seriousness
- Determination of relationship to investigational device and relationship to procedure
-)
- Description of adverse event outcome, if applicable
- Date and signature of investigator

7.5.4.2 Device Deficiency Form

The device deficiency form will contain the following information:

- Device lot or serial number, if applicable
- Date of deficiency
- Description of deficiency and actions taken
- Determination of adverse event (if yes, the AE form shall be completed)
- Determination if Device Deficiency might have led to a SAE if a) suitable action had not been taken or b) intervention had not been made or c) if circumstances had been less fortunate (if yes, the AE form must be faxed to the sponsor)
- Date and signature of investigator

CONFIDENTIAL - THIS DOCUMENT CONTAINS CONFIDENTIAL AND PROPRIETARY INFORMATION. THIS DOCUMENT MAY NOT BE REPRODUCED, USED, OR DISCLOSED TO ANY THIRD PARTY WITHOUT PRIOR WRITTEN CONSENT.

7.6 Suspension or Premature Termination of the Clinical Investigation

In the event of unforeseen or increased risks to subjects encountered during the course of the study, the sponsor may decide to suspend or prematurely terminate the clinical study. Such decision would be made after discussion with the Principal Investigator.

The sponsor may also decide to terminate the study for other reasons. The same procedure will be followed for whichever the reason for termination and is explained below.

Discontinuation shall be affected by fax or registered mail. If discontinuation occurs, the Competent Authority and the Ethics Committees involved shall be informed in writing within 15 calendar days after the discontinuation has been notified to the Investigator.

If suspension or premature termination occurs

1. The Sponsor shall remain responsible for providing resources to *fulfil* the obligations from the CIP and existing agreements for following up the subjects enrolled in the clinical investigation, and

2. The Principal Investigator or authorized designee shall promptly inform the enrolled subjects at his/her investigation site, if appropriate.

The termination of clinical investigation shall be notified to Competent Authority by sending an official signed letter notifying the end of the study. This can be done by email through ct.rd@famhp.be with the following subject "end of clinical investigation – 80M0XXX"

7.7 Monitoring Plan

The Sponsor will be allowed to have access to all source documents needed to verify the entries in the CRFs and other CIP-related documents provided that subject confidentiality is maintained in agreement with applicable laws.

It will be the Sponsor's responsibility to inspect the CRFs at regular intervals throughout the study, to verify the adherence to the CIP and the completeness, consistency and accuracy of the data being entered on them. The monitoring standards require full verification for the presence of informed consent, adherence to the inclusion/exclusion criteria, documentation of SAEs/SADEs and the recording of the main efficacy, safety, and performance endpoints.

Once the study starts, an initiation visit will be performed at the study site. A routine visit will be performed every 2 weeks and a final visit will be performed after the last patient had been explanted and finished all study procedures. During each monitoring visit, 100% of new data will be checked. TRIUM will perform monitoring activities according to TRIUM SOP's on monitoring activities. Each visit will be documented in a form of monitoring report.

Depending on the quality of the data, additional monitoring visits may be necessary according to the sponsor's discretion.

8 Statistical Considerations

No statistical analysis will be performed for this early feasibility clinical investigation.

8.1 Justification of Sample Size

Since sample size for the early feasibility study does not have to be statistically driven, it is therefore justified to perform a clinical investigation with 7 participants. It is possible to collect large sample of data sets (raw NRI spectra of ketones, lactate and glucose) from small sample of study subjects. Moreover, 7 subjects are judged to be enough to confirm the preliminary safety of YANG SYSTEM.

CONFIDENTIAL - THIS DOCUMENT CONTAINS CONFIDENTIAL AND PROPRIETARY INFORMATION. THIS DOCUMENT MAY NOT BE REPRODUCED, USED, OR DISCLOSED TO ANY THIRD PARTY WITHOUT PRIOR WRITTEN CONSENT.

9 Data Management

The clinical data manager working on behalf of Indigo Diabetes N.V. will be responsible to perform all data management activities. A study specific Data Management Plan (DMP) including all necessary information concerning data entry and data cleaning will be written.

9.1 Data Management Plan

Case Report Form (CRF) data collected will be subject to data quality control measures in compliance with all applicable data management procedures. Edit checks will be applied to identify discrepant data. All CRFs will be audited against corresponding database output to ensure accuracy. Data management documentation will be generated and maintained throughout the process.

Data management staff will check that all data in the CRFs are correctly entered into the study database. Data management personnel will correct obvious errors, and a query will be sent to the CRA for confirmation of the correction by the investigator. Other errors or omissions will also be sent to the CRA for resolution by the investigator, using data management standard data query forms. The CRFs will be corrected as required, and the corrected data entered into the database. The copy of the signed query form will be filed with the CRFs.

Prior to database lock, the database will be validated. When all queries have been resolved, the database will be locked. Any changes to the database after that time will require joint written agreement between the Investigator and Sponsor.

9.2 Data Collection and Entry

Investigator will compile all clinical data in the electronic CRF. The investigator will assign patient-ID to each study subject. On the CRF no patient name shall be indicated, only the patient ID shall be used to reference the case. Investigator will keep confidential a list of the subjects' names linked to their ID number.

The CRFs will be reviewed and checked during the monitoring visits. If discrepancies are found, changes can be made by the investigator applying good recording practices (any change or correction to data reported in CRF shall be dated, initialled and explained if necessary, and shall not obscure the original entry).

Upon entry, the data will be checked for consistency and completeness. If deficiencies are identified, data queries will be sent to the investigator. The data query will contain the following information:

Query part

- CRF identification
- Date of query
- Description of query

Response part

- Response to query
- Date and signature of investigator.

9.3 Data Verification

After all queries have been resolved, the data entered into the reporting form will be verified by the CRA. This verification will be documented in the eCRF. Monitoring visits will be performed to allow source data verification. The focus of this verification will be towards the primary endpoints and safety data (adverse event reporting). A risk-based approach will be implemented.

9.4 Data Cleaning

In a multistage procedure, the data obtained will be checked for their plausibility and consistency. Any inconsistencies, which may be detected and missing or implausible data, will be queried and clarified, and necessary changes will be

CONFIDENTIAL - THIS DOCUMENT CONTAINS CONFIDENTIAL AND PROPRIETARY INFORMATION. THIS DOCUMENT MAY NOT BE REPRODUCED, USED, OR DISCLOSED TO ANY THIRD PARTY WITHOUT PRIOR WRITTEN CONSENT.

carried out. The traceability of changes in valid data or system operations will be documented by an audit trail. The audit trail will start after the first data entry step.

9.5 Data Analysis

The collected data will serve only for the explanatory purpose and will be used to develop the next version of YANG SENSOR. The safety related endpoints will be analysed in order to confirm safe integration of hardware in human subcutaneous tissue and to confirm safety of surgical technique for implantation and explantation of YANG SENSOR

All recorded and derived variables will be presented using appropriate descriptive summary statistics (continuous and ranked data: sample size, mean, standard deviation, minimum, first quartile, median, third quartile, maximum; categorical data: sample size, absolute and relative frequency).

All adverse events with onset during the study period will be displayed in summary tables. Tables will show the number of adverse events, the number and the percentage of patients affected by relation to implantation, explantation, device and underlying disease.

9.6 Data Retention

All study data at sponsor and investigation sites will be kept for **15 years** after study termination.

9.7 Protection of Patient's Privacy

The patient privacy will be protected at all time. To do so, the CRF will not contain the information on identity of the patient but only his/her patient ID and his/her initials specifically defined for this study. The principal investigator will maintain a list containing all patients indentification. The initials are used for controls. The list will be kept confidential by the principal investigator.

10 Ethical and Legal Aspects

For the clinical investigation described in this CIP applicable national legal provisions including ethical procedures will be respected. Administrative Procedures must be done according to national laws and requirements.

11 Data Quality Assurance (Auditing)

Throughout every part of the clinical investigation the quality management system of Indigo Diabetes N.V. will be applied. Investigator site and study documentation may be subject to Quality Assurance audits during the course of the study. In addition, regulatory bodies at their discretion may conduct inspections, during and after study completion.

12 Amendments to this Study Plan

In case the clinical investigation plan needs to be amended, a revision of the plan will be performed. The version number of the plan will be incremented (1.1, 1.2, 2.0, 3.0, etc.) and the same persons (or functions if change(s) in the team occurred) that approved the original plan will approve the new revision.

Any new revision will be submitted by the investigators to the Ethics Committees for approval.

Any new revision will be submitted by the Sponsor to the Competent Authority; The Federal Agency for Medicines and Health Products of Belgium (FAMHP) for approval if substantial changes are performed to the study design or documents. Any new revision of the documentation that does not contain substantial changes will also be submitted to the Competent Authority for notification purpose.

CONFIDENTIAL - THIS DOCUMENT CONTAINS CONFIDENTIAL AND PROPRIETARY INFORMATION. THIS DOCUMENT MAY NOT BE REPRODUCED, USED, OR DISCLOSED TO ANY THIRD PARTY WITHOUT PRIOR WRITTEN CONSENT.

The revised clinical investigation plan will be made effective only after necessary approvals above have been obtained.

13 Deviations from the Clinical Investigation Plan

As a general rule, the investigator is not allowed to deviate from the clinical investigation plan, except under emergency circumstances to protect the rights, safety and well-being of the subjects.

All deviations will be recorded in a specific deviation form, which is part of the complete case report form package. The deviation form will capture the following information:

- Patient identification
- Date of deviation
- Description of deviation
- Actions taken to mitigate the effects of the deviation
- Determination if the deviation affects subject's rights, safety and wellbeing, or the scientific integrity of the clinical investigation
- Date and signature of investigator

All deviation forms will be reviewed by the Sponsor Representative during the monitoring visits. If new deviations are identified during such visits, the corresponding deviation forms will be created.

All deviations that affect subject's rights, safety and wellbeing, or the scientific integrity of the clinical investigation shall be reported by the Investigator to his Ethics Committee and to the Sponsor immediately but not later than within 3 calendar days after the occurrence of the deviation. Such deviation shall be reported to the Sponsor with no delay.

14 Financial Plan and Costs

This clinical investigation is funded by Indigo Diabetes N.V., Bollebergen 2B box 5, 9000 Gent, Belgium. An investigation contract has been established between the investigation site and the Sponsor that regulates the financial aspects of this study. A mark-up according to UZA contract guidelines of October 1st, 2019 is paid to the institution to cover all the additional costs generated by this study.

The study participants will be financially reimbursed for the participation in the trial (the expenses including transportation and time will be refunded in amount of 800 Euro per day for subjects in the “diabetes cohort” (up to 8000 Euro for completing all scheduled study visits) and 600 Euro per day for subjects in the “healthy subject cohort” (up to 6000Euro for completing all scheduled visits. The difference between the 2 groups is based on risk profile and differential intensity of the study tests/procedures.

15 Publication Policy

Within 30 days after the final completion of the clinical investigation, a full Final Report will be written. The Principal Investigator will be asked to review and sign the Final Report.

The findings of this study may be published by the Sponsor or Investigator in a scientific journal and presented at scientific meetings. The manuscript will be circulated to the Investigator before submission. Confidentiality of subjects in reports/publications will be guaranteed. The precise procedures for publication are described in Clinical Study Agreement.

CONFIDENTIAL - THIS DOCUMENT CONTAINS CONFIDENTIAL AND PROPRIETARY INFORMATION. THIS DOCUMENT MAY NOT BE REPRODUCED, USED, OR DISCLOSED TO ANY THIRD PARTY WITHOUT PRIOR WRITTEN CONSENT.

16 Conflict of Interest

The trial is initiated and fully financed by Indigo Diabetes N.V. The Principal Investigator Prof. Dr. med. Christophe De Block is not affiliated with the Sponsor and has full scientific integrity. The investigation site receives a fixed amount of money for each patient completing the trial.

17 Statement of Compliance

The Principal Investigator and the Sponsor certify that:

- The clinical investigation will be conducted in accordance with the ethical principles that have their origin in the Declaration of Helsinki.
- The clinical investigation shall be conducted in full compliance with the device EU Council Directive 90/385/EEC (Active Implantable Medical Device Directive; AIMDD)s, Belgian Royal Decree dated July 15, 1997 governing the active implantable medical devices, Belgian National Law dated 7th May 2004 related to experiments on human people and with international standards ISO 14155: 2020
- The clinical investigation shall not begin until the approvals from the Ethics Committees competent and from the Regulatory Authority; The Federal Agency for Medicines and Health Products of Belgium (FAMHP) have been obtained.
- Any additional requirements imposed by the Ethics Committees or by the Regulatory Authority in their approval letter will be followed.
- The clinical investigation will be registered in a publicly accessible database (e.g. www.clinicaltrials.gov) before recruitment of the first subject.
- The Sponsor; Indigo Diabetes N.V., Bollebergen 2B box 5, 9000 Gent, Belgium, has contracted an insurance to cover any subject's incapacitation resulting from damage to their health attributable to the investigation.

18 Bibliography

1. Yeung, K. T. D., Reddy, M. & Purkayastha, S. Surgical options for glycaemic control in Type 1 diabetes. *Diabet. Med.* **36**, 414–423 (2019).
2. Aleppo, G. & Webb, K. Continuous Glucose Monitoring Integration in Clinical Practice: A Stepped Guide to Data Review and Interpretation. *J. Diabetes Sci. Technol.* **13**, 664–673 (2019).
3. Beck, R. W., Bergenstal, R. M., Laffel, L. M. & Pickup, J. C. Advances in technology for management of type 1 diabetes. *Lancet (London, England)* **394**, 1265–1273 (2019).
4. Levine, B. J., Close, K. L. & Gabbay, R. A. Reviewing U.S. Connected Diabetes Care: The Newest Member of the Team. *Diabetes Technol. Ther.* **22**, 1–9 (2019).
5. Zimmerman, C., Albanese-O'Neill, A. & Haller, M. J. Advances in Type 1 Diabetes Technology Over the Last Decade. *Eur. Endocrinol.* **15**, 70–76 (2019).
6. Grant, A. K. & Golden, L. Technological Advancements in the Management of Type 2 Diabetes. *Curr. Diab. Rep.* **19**, 163 (2019).
7. Akturk, H. K. & Garg, S. Technological advances shaping diabetes care. *Curr. Opin. Endocrinol. Diabetes. Obes.* **26**, 84–89 (2019).
8. Ajjan, R., Slattery, D. & Wright, E. Continuous Glucose Monitoring: A Brief Review for Primary Care Practitioners. *Adv. Ther.* **36**, 579–596 (2019).
9. Polonsky, W. H. Psychosocial Aspects of Diabetes Technology: Adult Perspective. *Endocrinol. Metab. Clin. North Am.* **49**, 143–155 (2020).
10. Kim, J., Campbell, A. S., de Ávila, B. E.-F. & Wang, J. Wearable biosensors for healthcare monitoring. *Nat. Biotechnol.* **37**, 389–406 (2019).

CONFIDENTIAL - THIS DOCUMENT CONTAINS CONFIDENTIAL AND PROPRIETARY INFORMATION. THIS DOCUMENT MAY NOT BE REPRODUCED, USED, OR DISCLOSED TO ANY THIRD PARTY WITHOUT PRIOR WRITTEN CONSENT.

Indigo Diabetes N.V
Bollebergen 2B box 5,
9000 Gent, Belgium
https://indigomed.com/

11. De Ridder, F., den Brinker, M. & De Block, C. The road from intermittently scanned glucose monitoring to hybrid closed-loop systems: Part A. Keys to success: subject profiles, choice of systems, education. *Ther. Adv. Endocrinol. Metab.* **10**, 2042018819865399 (2019).

12. Barnard, K. D. *et al.* Acceptability of Implantable Continuous Glucose Monitoring Sensor. *J. Diabetes Sci. Technol.* **12**, 634–638 (2018).

CONFIDENTIAL - THIS DOCUMENT CONTAINS CONFIDENTIAL AND PROPRIETARY INFORMATION. THIS DOCUMENT MAY NOT BE REPRODUCED, USED, OR DISCLOSED TO ANY THIRD PARTY WITHOUT PRIOR WRITTEN CONSENT.

APPENDIX I: Revision History

This CIP may be amended as appropriate by the Sponsor. Rationale will be included with each amended version in the revision history table below. The version and date of the amendments will be documented.

EC and relevant Regulatory Authorities, if applicable, will be notified of amendments of the CIP.

Version	Date	Details	Rationale
1.0	17SEP2020	First release of CIP	NA
2.0	30MAR2021	Sample frequency changes	For each sample, maximum 0.5 ml of blood is used for analysis. However, an extra maximum 1.5 ml of catheter liquid is required as "waste" (immediately discarded), to obtain a clean reference measurement. With an average of 100 blood samples per day, the original protocol would result in too much blood loss over the 30-day period of the study. As the results from the initial 3 patients indicate that samples obtained every 5 minutes (or every 10 minutes) are sufficient to perform the analysis, both subject safety and study validity can be assured with the reduced number of samples.
		Removal of Medtronic CGM	To reflect that only a Dexcom G6 device will be used as commercially available GCM device
		Breath analyser	To measure the alcohol levels
		Ultrasound imaging	to evaluate the depth of the sensor and the encapsulation, if any
		Inclusion criteria for healthy volunteers – able to perform intense physical activity	To reach high lactate values, intense physical activity is required
		Vulnerable population	Additional ICF addendum for sponsor employees participating in the study
		Treatment allocation	No specific order. At investigator's discretion
		Interferent start	The excursion of interferents should only start after the initial 'warm-up' period (1 hour) of the YANG SENSOR.
		Max. 2 hours of biking	The lactate profile needs to build up over a longer period of time, to ensure a more gradual increase in lactate, and a better match between lactate values in blood and interstitial fluid.
		Biking exercise start for T1DM at glucose CGM values in the range of 150-180 mg/dL.	Avoid the T1DM subject going into hypo- or hyperglycemia during the exercise.
		Max 6-8 high alcohol containing beverages intake	In order to achieve the predefined values of 90 mg/dl, a larger quantity of alcohol consumption than expected is required (and taking into account individual response to alcohol intake)
		The subject will alternate a low ethanol day with a high ethanol day	To make the measurement days more comfortable for the subject and avoid discomfort.
		Timepoint of confounder administration around glucose value of 185 mg/dl for subject 2	The administration of confounders should only start after the hyperglycemia, to make sure the long-term

CONFIDENTIAL - THIS DOCUMENT CONTAINS CONFIDENTIAL AND PROPRIETARY INFORMATION. THIS DOCUMENT MAY NOT BE REPRODUCED, USED, OR DISCLOSED TO ANY THIRD PARTY WITHOUT PRIOR WRITTEN CONSENT.

		and 300 mg/dl for subjects 1, 3 and 4	data analysis is not impacted by any side effects of the confounders (e.g., rapid temperature changes).
		60 ml ketone drink for healthy volunteers	General amount of 60 ml ketone drink unrelated to the weight for healthy volunteers to raise the ketone levels in blood
		More variation between visits for subject 3 with regards to the ketone intake and low carb diet.	This lowers the burden for the subject and will give an interesting dataset with a different relationship between glucose-temperature-interferent.
		Removal of physical examination requirement at implantation visit	Per discussion with site and sponsor, there is no added value in collecting these data at implantation visit.
		Updates in the Adverse Event, Adverse Device Effects and Device Deficiencies section	To align all definitions and wording to the ISO 14155 standards and MEDDEV 2.7/3 guidelines
		Minor corrections (mainly for typo's) and clarifications throughout the CIP	NA

CONFIDENTIAL - THIS DOCUMENT CONTAINS CONFIDENTIAL AND PROPRIETARY INFORMATION. THIS DOCUMENT MAY NOT BE REPRODUCED, USED, OR DISCLOSED TO ANY THIRD PARTY WITHOUT PRIOR WRITTEN CONSENT.
