# Supplementary material for: Early feasibility study with an implantable near-infrared spectroscopy sensor for glucose, ketones, lactate and ethanol
Source: PLoS One. 2024 May 3;19(5):e0301041. doi: 10.1371/journal.pone.0301041 (PMC11068174; doi:10.1371/journal.pone.0301041)
Supplement: Attachment — Submitted filename: renamed_898d1.docx [file pone.0301041.s006.docx]

# COMMENTS – Manuscript PONE-D-23-13677

Title: “Early feasibility study with an implantable near-infrared spectroscopy sensor for glucose, ketones, lactate and ethanol”

**Important note: This review pertains only to ‘statistical aspects’ of the study and so ‘clinical aspects’ [like medical importance, relevance of the study, ‘clinical significance and implication(s)’ of the whole study, etc.] are to be evaluated [should be assessed] separately/independently. Further please note that any ‘statistical review’ is generally done under the assumption that (such) study specific methodological [as well as execution] issues are perfectly taken care of by the investigator(s). This review is not an exception to that and so does not cover clinical aspects {however, seldom comments are made only if those issues are intimately / scientifically related & intermingle with ‘statistical aspects’ of the study}. Agreed that ‘statistical methods’ are used as just tools here, however, they are vital part of methodology [and so should be given due importance]. I look at the manuscript in/with statistical view point, other reviewer(s) look(s) at it with different angle so that in totality the review is very comprehensive. However, there should be efforts from authors side to improve (may be by taking clues from reviewer’s comments). Therefore, please do not limit the revision only (with respect) to comments made here.**

**COMMENTS:** Although this manuscript is well drafted [and the study is excellent with respect to most of the aspects], I have few observations/concerns (different opinion) which are given below:

Agreed that this study being ‘pilot’ (feasibility study) in nature, sample size is not a big issue. However, considering the variation, sample size seems to be too small [7 participants (4 with type 1 diabetes (T1D), 3 healthy volunteers)]. I can understand that ‘Implantation’ of the sensor & six 8-hour long measurement visits are are not easy / cumbersome.

It is true [as is often quoted] that “Pilot (Proof of Concept/feasibility) studies typically involve a small number of subjects, as well as more latitude [i.e., leeway, freedom, liberty] in statistical requirements.”], in my opinion, methodological issues need to be very rigorous followed {like in case of clinical trial, CONSORT guidelines are to be strictly observed/followed}. You may definitely know that CONSORT guidelines for Pilot trial(s) is available.

Moreover, I request these learned authors to discuss the appropriateness of choice of [Healthy volunteers as] ‘control’ group and also the dis-similar handling of this group (refer to ‘Healthy volunteers’ section in lines 238 onwards) with well experienced & well qualified Biostatistician. This suggestion because I doubt about (the validity of) both these points. For ‘pilot/feasibility study’ it hardly matters / is definitely not an important issue [because even single group may suffice]. However, this knowledge will be helpful while planning a big/main trial/study.

Except these minor points, the article is acceptable. However, mind you that as pointed out in ‘important note’ above “This review pertains only to ‘statistical aspects’ of the study and so ‘clinical aspects’ should be assessed separately/independently. ‘Minor Revision’ is recommended.
