# Supplementary material for: Early feasibility study with an implantable near-infrared spectroscopy sensor for glucose, ketones, lactate and ethanol
Source: PLoS One. 2024 May 3;19(5):e0301041. doi: 10.1371/journal.pone.0301041 (PMC11068174; doi:10.1371/journal.pone.0301041)
Supplement: Attachment — Submitted filename: Glow_PlosOne_Answers to Reviewers.docx [file pone.0301041.s007.docx]

***De Ridder F et al. et al.***

*Early feasibility study with an implantable near-infrared spectroscopy sensor for glucose, ketones, lactate and ethanol*

Manuscript Id**: PONE-D-23-13677**

# Answers to Reviewers

## Reviewer 1

1. “I would suggest adding cannabis as a reason for DKA and for ketone monitoring in introduction, that is a main issue in USA. You can find an article to cite in JAMA Internal Medicine, Diabetes Care etc”
   Thank you for the suggestion, we added cannabis use to the list of risk factors for diabetic ketoacidosis, citing the findings on Kinney et al, Diabetes Care 2020 on line 78 of the revised manuscript.
2. “You can add the high cost of blood ketone test strips compared to urine test strips in your introduction. Another legit reason and limitation.”
   In response to this suggestion, we updated the reasons for limited ketone testing in the discussion section of the paper (line 488 and following) as follows: “but yet many people with T1D do not even own home ketone monitoring strips, possibly due to the high cost of these strips, and in one third of cases these strips have exceeded their expiry date.”
3. “line 124, 236, 299, 360, 397, 410, 417, 421, there is an error of citation, unintended words.”
   Cross-references to the tables and figures were not rendered correctly by the manuscript submission system. In the revised version, a version without field codes will be uploaded to remediate this.
4. “Did you use US in 4 subjects because you could not locate the sensor or just for another part of the study? Were all sensor palpable at the end of the study?”
   The ultrasound evaluation was planned in the study design as part of the safety and biocompatibility endpoint of the study. In view of its size, there were no issues locating or retrieving the sensors at the time of explantation. A statement to this effect was added to the results section of the revised manuscript (line 335). All sensors were indeed easily palpable at the end of the implantation period.
5. “Is there a way to let it heal without any stiches like Eversense procedure? I understand this is a bigger sensor about 3 cm, I am asking for future practicality point.”
   The subcutaneous layer needs to be closed with absorbable sutures. The skin wound is not a superficial small wound where wound tape, e.g. Steri-Strips (3M), could be solely used without any skin stitches. It is however a straight clean wound where wound tape could be used for wounds even over 5 cm, but they do not support lacerations well in lax skin or wounds that are under a lot of tension. Surgical glue, e.g. DERMABOND, could also be an option to close the skin. The functional tensile strength of DERMABOND is comparable to that of 5–0 sutures, however, because the first-day strength of DERMABOND is much less than that of sutures, application is limited to wounds on the face, extremities and selected areas over the torso. This modality has as disadvantage that it is brittle and subject to fracture when used on long lacerations or over skin creases. In conclusion, the wound does not have the optimal location and size to use wound tape or surgical glue, but future reduction of sensor dimensions could allow alternative wound closure techniques.
   In response to this comment, the following statement was added to the discussion section of the paper (line 426 and following): “The current dimensions of the implantable sensor, as used in the GLOW study, require wound closure by means of absorbable sutures (or equivalent). Future design considerations include the reduction of sensor dimensions which could allow more practical wound closure.”
6. “How long did patients with T1D stay in hypo and hyper challenge?”
   The measurement visit was planned over the full day. The patients went gradually from normoglycemia to hyperglycemia (250 or 400 mg/dl for lactate and non- lactate challenge resp), then to hypoglycemia (40 mg/dl) and back to normoglycemia state. Per protocol the max rate of change for decreasing glucose levels was 100 mg/dl/hour.
   The time spent in hypoglycaemia was documented, although it was not part of the outcome parameters of the study.
   On average people spent 22 minutes in hypoglycemia (< 54 mg/dl), ranging between 9-32 minutes. When a glucose level of 40 mg/dl was reached, patients were immediately given 15 ml of a soft drink (cola).
   The mean time spent in hyperglycemia (> 180 mg/dl) was 144 minutes, varying between subjects from 80 to 221 minutes.
   The mean time spent to go from the hyperglycemic maximum target (250 or 400 mg/dl for lactate and non-lactate challenge resp) to a glucose level of 40 mg/dl was 232 minutes (varying between 172-290 minutes).
7. “To add interfering substance in discussion for Eversense, mannitol is also to add for interference in addition to tetracycline.”
   Mannitol was added as a potential interfering compound for the Eversense sensor on line 448 of the revised manuscript.

Suggestions

1. “How about using Akturk’s method (PMID: 32031415, PMID: 33543901) using NIR to find implanted sensors instead of US? You should have a plan if they get lost, especially planning a sensor for 2 years to be in.”
   The authors thank the reviewer for this suggestion. In this study, there was no issue locating the implants. In view of the size of the implant (3 x 0.7 x 1.5 cm) and the subcutaneous implantation in the abdominal region, we do not expect any issues retrieving the sensors. It is good to know, however, that alternative localization methods exist. A statement that there were no issues locating the sensors for explantation was added to the revised manuscript (line 335).
2. “What is the reason to choose the location site vs other sites like arm etc.”
   The abdominal region was chosen as the site of implantation because it provides enough space for the implantation and is easily reachable for data exchange with the current sensor and the requirements for charging the sensor and communicating with the sensor. Other implant locations can be evaluated if design aspect of the future sensor will allow. Indeed, taking into account the size of this sensor, which is a prototype, the arm would not be a suitable site.
3. “There is no consensus about what the threshold for ketone in T1D should be, different guidelines, expert opinions suggest different things just to be aware.”
   Early identification of ketosis could facilitate preemptive measures to prevent impending ketoacidosis. A beta-hydroxybutyrate level < 0.6 mmol/L is generally accepted as posing no particular increased risk (cfr references listed below). For ketone levels between 0.6-1.0 mmol/L, the Australian Diabetes Educators Association (ADEA) advises that blood ketones should be checked every 2 hours, and every hour for ketone levels >1.0 mmol/L For sick day management, the American Diabetes Association (ADA) guidelines to parents of children with T1D advises to “check blood or urine ketones as often as every 4 hours,”.

- Australian Diabetes Educators Association. Clinical Guiding Principles for Sick Day Management of Adults with Type 1 and Type 2 Diabetes. Technical document. Canberra: Australian Diabetes Educators Association; 2016.
- American Diabetes Association. Checking For Ketones. Available at: [http://www.diabetes.org/living-with-diabetes /treatment-and-care/blood-glucose-control/checking-for-ketones.html](http://www.diabetes.org/living-with-diabetes%20/treatment-and-care/blood-glucose-control/checking-for-ketones.html)
- Lee MH, Paldus B, Krishnamurthy B, McAuley SA, Shah R, Jenkins AJ, et al. The Clinical Case for the Integration of a Ketone Sensor as Part of a Closed Loop Insulin Pump System. J Diabetes Sci Technol. 2019;13:967–73. <https://doi.org.10.1177/1932296818822986> (cited in the manuscript as reference 14).
- Nguyen KT, Xu NY, Zhang JY, Shang T, Basu A, Bergenstal RM, et al. Continuous Ketone Monitoring Consensus Report 2021. J Diabetes Sci Technol 2021;19322968211042656. <https://doi.org.10.1177/19322968211042656> (cited in the manuscript as reference 13).

4. “Consider high ketone levels in future studies like healthy volunteers eat strict keto and T1D using SGLT2.”
   The increased risk of people with T1D using SGLT2i is mentioned in the discussion section of the manuscript. Since this is an early feasibility study and the sensor is currently intended for patients with diabetes, we consider measuring ketones in healthy persons following a ketogenic diet out of scope for this study. It will be considered as a potential additional application of the device in the future, however.

## Reviewer 2

1. “Agreed that this study being ‘pilot’ (feasibility study) in nature, sample size is not a big issue. However, considering the variation, sample size seems to be too small [7 participants (4 with type 1 diabetes (T1D), 3 healthy volunteers)]. I can understand that ‘Implantation’ of the sensor & six 8-hour long measurement visits are not easy / cumbersome.”
   Although the number of subjects is small, the number of datapoints obtained in this study is not: over the 6 measurement visits of 8 hours, the continuous measurements of the sensor were compared with values from 60-70 blood samples obtained every 5-10 minutes.
2. “It is true [as is often quoted] that “Pilot (Proof of Concept/feasibility) studies typically involve a small number of subjects, as well as more latitude [i.e., leeway, freedom, liberty] in statistical requirements.”], in my opinion, methodological issues need to be very rigorous followed {like in case of clinical trial, CONSORT guidelines are to be strictly observed/followed}. You may definitely know that CONSORT guidelines for Pilot trial(s) is available.”
   The CONSORT guidelines are intended to improve the reporting of randomized trials and as such are not applicable to our study, which is not a randomized trial. The primary endpoint was to investigate the safety and biocompatibility of the sensor over a 28-day implantation period, the secondary endpoint to study the parameters of the implantation and explantation procedure, whereas the measurements of glucose, glucose, β-hydroxybutyrate, lactate and ethanol were only explorative endpoints. Larger follow-up trials taking into account statistical sample size considerations are being conducted at present.
3. “Moreover, I request these learned authors to discuss the appropriateness of choice of [Healthy volunteers as] ‘control’ group and also the dis-similar handling of this group (refer to ‘Healthy volunteers’ section in lines 238 onwards) with well experienced & well qualified Biostatistician. This suggestion because I doubt about (the validity of) both these points. For ‘pilot/feasibility study’ it hardly matters / is definitely not an important issue [because even single group may suffice]. However, this knowledge will be helpful while planning a big/main trial/study.”
   It was not the objective of this study to “compare” persons with T1D and healthy volunteers. The reason not to perform this early feasibility study only in healthy volunteers was to already obtain safety data in persons with T1D, and to allow for measurements of glucose across the whole relevant clinical range, which would not be possible in healthy volunteers.
