# Supplementary material for: Early feasibility study with an implantable near-infrared spectroscopy sensor for glucose, ketones, lactate and ethanol
Source: PLoS One. 2024 May 3;19(5):e0301041. doi: 10.1371/journal.pone.0301041 (PMC11068174; doi:10.1371/journal.pone.0301041)
Supplement: Attachment — Submitted filename: Glow_PlosOne_Answers to Reviewers_R2.docx [file pone.0301041.s008.docx]

***De Ridder F et al. et al.***

Early feasibility study with an implantable near-infrared spectroscopy sensor for glucose, ketones, lactate and ethanol

Manuscript Id: **PONE-D-23-13677R1**

# Answers to Reviewers

## Reviewer 1

No comments

## Reviewer 2

1. Few of the comments made on first draft were addressed, however, frankly speaking, I am/was not very much convinced (or happy) for reasons given or arguments made.
   While answering my second comment you said “The CONSORT guidelines are intended to improve the reporting of randomized trials and as such are not applicable to our study, which is not a randomized trial” which is surprising. As you know, all ‘Clinical Trials’ [random or non-random] must follow Consolidated Standards of Reporting Trials (CONSORT) guidelines [letter ‘R’ in CONSORT stands for ‘Reporting’ and not for ‘Random’]. Since your article type is ‘Clinical Trial’, you are supposed to cover at least important items in CONSORT in the report (which is not seen). You may be aware of one article on guidelines for reporting non-randomised studies {Reeves BC, Gaus W. ‘Guidelines for reporting non-randomised studies’, Forsch Komplementarmed Klass Naturheilkd 2004 Aug;11 Suppl 1:46-52. doi: 10.1159/000080576}. Non-randomised studies (NRSs) are more susceptible to bias. CONSORT has improved the reporting of key information, highlighting missing key information for users. Researchers have a responsibility to report essential information that allows users to assess the susceptibility of NRS to selection, performance, detection and attrition bias.
   I feel, ‘let the respected editor decide the future course’ and I do not have any specific recommendation as such [though only as system requirement, I choose reject].
   We agree with the reviewer that rigorous reporting methodology is of key importance. Please find the filled out CONSORT checklist below (This information is also uploaded as a supplemental information file in the editorial manager application).
   We wish to additionally point out that the PlosOne Editorial Office requested us to provide a filled out CONSORT **or** TREND reporting checklist. The latter we already added to our submission in May 2023. The TREND checklist has the same purpose and contains more or less the same information as the CONSORT checklist but is intended for non-randomized studies.
   Please also take note of the fact that the Protocol and funding are not included in the manuscript as per the Journal’s instructions to authors. These elements are required for submission, however, and have been provided by us in the journal’s submission system. Both the funding statement and the study protocol will be published by PlosOne alongside the manuscript.

CONSORT 2010 checklist of information to include when reporting a randomised trial*

| Section/Topic | Item No | Checklist item | Reported on page No |
| --- | --- | --- | --- |
| Title and abstract | | | |
|  | 1a | Identification as a randomised trial in the title | Not Applicable (NA) |
|  | 1b | Structured summary of trial design, methods, results, and conclusions (for specific guidance see CONSORT for abstracts) | 2-3 |
| Introduction | | | |
| Background and objectives | 2a | Scientific background and explanation of rationale | 4-6 |
|  | 2b | Specific objectives or hypotheses | 8-9 |
| Methods | | | |
| Trial design | 3a | Description of trial design (such as parallel, factorial) including allocation ratio | 7-8 |
|  | 3b | Important changes to methods after trial commencement (such as eligibility criteria), with reasons | NA |
| Participants | 4a | Eligibility criteria for participants | 8 |
|  | 4b | Settings and locations where the data were collected | 7 |
| Interventions | 5 | The interventions for each group with sufficient details to allow replication, including how and when they were actually administered | 10-12 |
| Outcomes | 6a | Completely defined pre-specified primary and secondary outcome measures, including how and when they were assessed | 8-9 |
|  | 6b | Any changes to trial outcomes after the trial commenced, with reasons | NA |
| Sample size | 7a | How sample size was determined | NA |
|  | 7b | When applicable, explanation of any interim analyses and stopping guidelines | NA |
| Randomisation: |  |  |  |
| Sequence generation | 8a | Method used to generate the random allocation sequence | NA |
|  | 8b | Type of randomisation; details of any restriction (such as blocking and block size) | NA |
| Allocation concealment mechanism | 9 | Mechanism used to implement the random allocation sequence (such as sequentially numbered containers), describing any steps taken to conceal the sequence until interventions were assigned | NA |
| Implementation | 10 | Who generated the random allocation sequence, who enrolled participants, and who assigned participants to interventions | NA |
| Blinding | 11a | If done, who was blinded after assignment to interventions (for example, participants, care providers, those assessing outcomes) and how | NA |
|  | 11b | If relevant, description of the similarity of interventions | NA |
| Statistical methods | 12a | Statistical methods used to compare groups for primary and secondary outcomes | 12-13 |
|  | 12b | Methods for additional analyses, such as subgroup analyses and adjusted analyses | NA |
| Results | | | |
| Participant flow (a diagram is strongly recommended) | 13a | For each group, the numbers of participants who were randomly assigned, received intended treatment, and were analysed for the primary outcome | 14 |
|  | 13b | For each group, losses and exclusions after randomisation, together with reasons | NA |
| Recruitment | 14a | Dates defining the periods of recruitment and follow-up | 7 |
|  | 14b | Why the trial ended or was stopped | NA |
| Baseline data | 15 | A table showing baseline demographic and clinical characteristics for each group | 14 |
| Numbers analysed | 16 | For each group, number of participants (denominator) included in each analysis and whether the analysis was by original assigned groups | 14 |
| Outcomes and estimation | 17a | For each primary and secondary outcome, results for each group, and the estimated effect size and its precision (such as 95% confidence interval) | 17-19 |
|  | 17b | For binary outcomes, presentation of both absolute and relative effect sizes is recommended | NA |
| Ancillary analyses | 18 | Results of any other analyses performed, including subgroup analyses and adjusted analyses, distinguishing pre-specified from exploratory | NA |
| Harms | 19 | All important harms or unintended effects in each group (for specific guidance see CONSORT for harms) | 14-16 |
| Discussion | | | |
| Limitations | 20 | Trial limitations, addressing sources of potential bias, imprecision, and, if relevant, multiplicity of analyses | 21 |
| Generalisability | 21 | Generalisability (external validity, applicability) of the trial findings | 22-24 |
| Interpretation | 22 | Interpretation consistent with results, balancing benefits and harms, and considering other relevant evidence | 19-24 |
| Other information | | |  |
| Registration | 23 | Registration number and name of trial registry | 1 |
| Protocol | 24 | Where the full trial protocol can be accessed, if available | Included in publication |
| Funding | 25 | Sources of funding and other support (such as supply of drugs), role of funders | In submission record |

Citation: Schulz KF, Altman DG, Moher D, for the CONSORT Group. CONSORT 2010 Statement: updated guidelines for reporting parallel group randomised trials. BMC Medicine. 2010;8:18.
© 2010 Schulz et al. This is an Open Access article distributed under the terms of the Creative Commons Attribution License (<http://creativecommons.org/licenses/by/2.0>), which permits unrestricted use, distribution, and reproduction in any medium, provided the original work is properly cited.

*We strongly recommend reading this statement in conjunction with the CONSORT 2010 Explanation and Elaboration for important clarifications on all the items. If relevant, we also recommend reading CONSORT extensions for cluster randomised trials, non-inferiority and equivalence trials, non-pharmacological treatments, herbal interventions, and pragmatic trials. Additional extensions are forthcoming: for those and for up-to-date references relevant to this checklist, see [www.consort-statement.org](http://www.consort-statement.org).
